# New vascular plant records for the Canadian Arctic Archipelago

**DOI:** 10.3897/phytokeys.52.8721

**Published:** 2015-06-25

**Authors:** Lynn J. Gillespie, Jeffery M. Saarela, Paul C. Sokoloff, Roger D. Bull

**Affiliations:** 1 Botany Section & Centre for Arctic Knowledge and Exploration, Research and Collections, Canadian Museum of Nature, P.O. Box 3443 Stn. D, Ottawa, Ontario K1P 6P4, Canada

**Keywords:** Floristics, Nunavut, Northwest Territories, Victoria Island, Baffin Island, Amaranthaceae, Juncaginaceae, Pteridaceae, *Cryptogramma*, *Platanthera*, *Suaeda*, *Triglochin*, *Utricularia*

## Abstract

The Canadian Arctic Archipelago is a vast region of approximately 1,420,000 km^2^, with a flora characterized by low species diversity, low endemicity, and little influence by alien species. New records of vascular plant species are documented here based on recent fieldwork on Victoria and Baffin Islands; additional records based on recent literature sources are mentioned. This paper serves as an update to the 2007 publication *Flora of the Canadian Arctic Archipelago*, and brings the total number of vascular plants for the region to 375 species and infraspecific taxa, an increase of 7.7%. Three families (Amaranthaceae, Juncaginaceae, Pteridaceae) and seven genera (*Cherleria* L., *Cryptogramma* R. Br., *Platanthera* Rich., *Sabulina* Rchb., *Suaeda* Forssk. ex J.F. Gmel., *Triglochin* L., *Utricularia* L.) are added to the flora, and one genus is deleted (*Minuartia* L.). Five species are first records for Nunavut (*Arenaria longipedunculata* Hultén, *Cryptogramma stelleri* (S.G. Gmel.) Prantl, *Puccinellia banksiensis* Consaul, *Saxifraga eschscholtzii* Sternb., *Utricularia ochroleuca* R.W. Hartm.)

## Introduction

**Figure 1.**
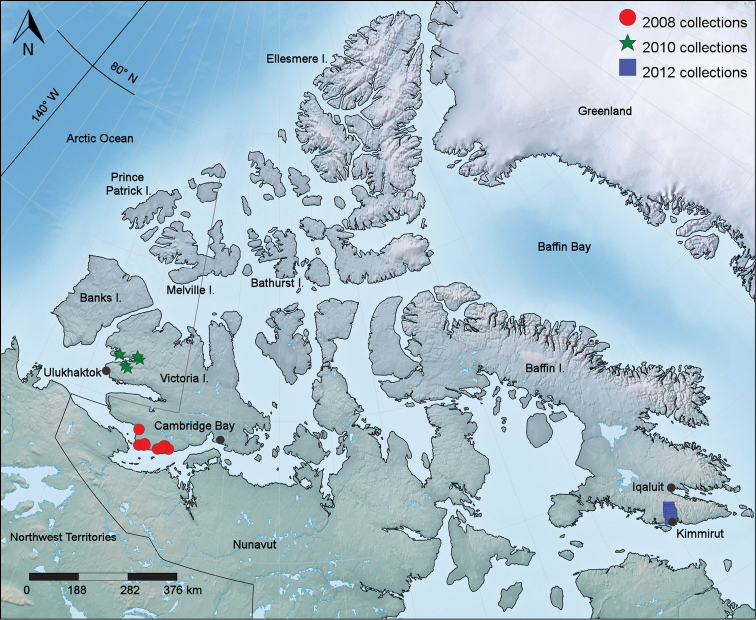
Map of the Canadian Arctic Archipelago showing 2008, 2010 and 2012 collection sites for new vascular plant records.

The Canadian Arctic Archipelago (CAA) is a group of islands occupying the northern third of Canada extending about 3000 km south to north and east to west, and covering approximately 1.42 million square kilometers. The archipelago comprises three very large islands, Baffin (507,451 km^2^), Victoria (217,291 km^2^), Ellesmere (196,236 km^2^), twelve islands between 10,000 and 71,000 km^2^, and many thousands of smaller islands (Fig. 1). Politically the majority of islands are within the territory of Nunavut, while the westernmost part of the CAA is within the Northwest Territories. During the last glacial maximum (LGM) ice sheets covered almost the entire area; today glaciers cover only about 11% of the land area (Sharp et al. 2014). The Arctic flora as a whole is characterized as a young flora with low species diversity, low endemicity, and is little influenced by alien species (Daniȅls et al. 2013). These characteristics are especially true for the flora of the CAA, which comprises only 349 recorded species and infraspecific taxa, no endemic species, and few, if any, stabilized alien species (Aiken et al. 2007).

Study of the Canadian Arctic Archipelago flora started with expeditions searching for a northwest passage in the early 1800s, such as the Parry Expedition (see Aiken et al. 2007 for a historical summary and list of collectors). Botanical collecting in this vast, difficult to access region continued sporadically through the 19^th^ and early 20^th^ centuries, mostly as part of large scientific expeditions. The first regional vascular plant floras appeared in the mid-1900s: Polunin’s (1940) *Botany of the Eastern Canadian Arctic* and Porsild’s (1955) *Vascular plants of the Western Canadian Arctic Archipelago*. Soon after, Porsild (1957, 1964) published a flora covering the entire CAA. In 2007 Aiken et al. published an updated flora for the CAA in digital format using Delta software.

Aiken et al. (2007) recorded and provided descriptions, maps and an interactive key for 349 species and infraspecific taxa (341 species plus eight subspecies) of vascular plants in the CAA, including three lycopods, eight monilophytes (ferns and *Equisetum* L.), and 338 flowering plants (no gymnosperms present). The genus *Papaver* L. was not fully treated at the time because it was undergoing revision; Solstad (2007) provided a provisional key of five species under the taxon entry *Papaver* spp., all of which are now recognized as distinct and occurring in the flora area (Elven et al. 2011). Counting these additional *Papaver* species (*Papaver cornwallisense* D. Löve, *Papaver dahlianum* Nordh., *Papaver labradoricum* (Fedded) Solstad & Elven, Papaver lapponicum subsp. occidentale (C.E. Lundstr.) Knaben, and *Papaver* sp. “Banks” [= *Papaver hultenii* Knaben]) brings the total to 353 species and infraspecific taxa (345 species), of which 342 are angiosperms. Aiken et al. (2007) comment in their Introduction that their flora “will provide a basis for much more research on Arctic plants in Canada in the coming years. There are many potentially very interesting areas that have never been botanised…. In these sites, certainly new records, as well as interesting new species to the Arctic Archipelago, are waiting to be discovered.”

Recent fieldwork by our team on Victoria Island in 2008 and 2010, and on Baffin Island in 2012, led to discoveries of species new to the CAA and many significant range extensions. Here we document our major findings, including species, genera and families new to the CAA and species new to the western and eastern parts of the Arctic Islands. A subset of these are first records of species for Nunavut. We also summarize the literature pertinent to the CAA flora published since 2007 (or that was not included in Aiken et al. 2007), including new species described, significant new records, and new records resulting from taxonomic and nomenclatural changes. This publication serves as an update to Aiken et al. (2007).

## Methods

Fieldwork in the CAA was carried out in 2008, 2010 and 2012. In July 2008 and 2010, we collected vascular plants on southern Victoria Island, Nunavut, and on north western Victoria Island, Northwest Territories (Fig. 1) (map generated with SimpleMappr; Shorthouse 2010). In July 2012 we collected along the Soper River in Katannilik Territorial Park and in the vicinity of Kimmirut (formerly Lake Harbour) on southern Baffin Island, Nunavut (Fig. 1). During these field seasons we collected 3021 vascular plant numbers, of which 81 are reported here as new records, representing 25 taxa. The first set of our collections is deposited in the National Herbarium of Canada (CAN), Canadian Museum of Nature. Duplicate specimens are deposited in ALA, ALTA, BABY, COCO, MICH, MO, MT, NYBG, O, UBC, US, UVIC, WIN, WTU (acronyms according to Thiers, continuously updated), as noted in the specimen citations. All specimens cited have been seen, unless otherwise noted. Species accounts are organized by major clade (monilophytes, monocots, and eudicots), and then alphabetically by family, genus and species. Family-level classifications follow Smith et al. (2006) for monilophytes and Angiosperm Phylogeny Group III (2009) for angiosperms. Numerous literature sources were consulted for nomenclature at the species level and below, including the *Flora of the Canadian Arctic Archipelago* (Aiken et al. 2007), the *Flora of North America North of Mexico* (Flora of North America Editorial Committee 1993+), and the *Annotated Checklist of the Panarctic Flora (PAF): Vascular Plants* (Elven et al. 2011). English common names mostly follow the *Database of Vascular Plants of Canada (VASCAN)* (Brouillet et al. 2010+, Desmet and Brouillet 2013). Global species distributions are modified from Elven et al. (2011); they provided a summary of the main geographical distribution patterns but are not intended to be exhaustive. Images of CAN specimens cited under Specimens Examined are available on Figshare (http://figshare.com; see Appendix) and the Canadian Museum of Nature's collections online website (http://collections.nature.ca/en/Search).

## Results

The new discoveries described here plus new species and significant distribution records published recently bring the total number of vascular plant taxa in the CAA to 42 families, 141 genera and 375 species and infraspecific taxa (368 species). Table 1 provides a summary of these additions to the flora of the CAA since the publication of Aiken et al. (2007). Twenty species and infraspecific taxa are documented here as new to the CAA, representing a 7.7% increase in the number of species and infraspecific taxa recognized in Aiken et al. (2007).

**Table 1.** Vascular plant species new to the Canadian Arctic Archipelago (CAA) since the publication of Aiken et al. (2007). Records are based on field collections and literature sources. Species new to the CAA, western CAA, eastern CAA and Nunavut are given. New records for one adventive species, one species previously known from only one collection in the CAA, one recently described species, and confirmation of three species excluded by Aiken et al. (2007) are also included.

Family	Species	New to CAA	New to western CAA	New to eastern CAA	New to Nunavut	Other	Source
Pteridaceae	*Cryptogramma stelleri* (S.G. Gmel.) Prantl	X			X		Current study
Cyperaceae	*Carex bicolor* Bellardi ex All.		X				Current study
	Carex brunnescens (Pers.) Poir. subsp. brunnescens	X					Current study
	*Eriophorum brachyantherum* Trautv. & C.A. Mey.		X				Current study
Juncaceae	*Luzula wahlenbergii* Rupr.		X				Current study
Juncaginaceae	*Triglochin palustris* L.	X					Current study
Orchidaceae	*Corallorhiza trifida* Chatelain		X				Current study
	Platanthera obtusata (Banks ex Pursh) Lindl. subsp. obtusata	X					Current study
Poaceae	Calamagrostis stricta subsp. groenlandica (Schrank) Á. Löve					Confirmed for eastern CAA	Current study
	Hordeum jubatum L. subsp. jubatum					New records, adventive species	Current study
	Leymus innovatus subsp. velutinus (Bowden) Tzvelev					Confirmed for CAA	Mason et al. (1972), Porsild and Cody (1980), Barkworth (2007), current study
	Leymus mollis (Trin.) Pilg. subsp. mollis	X					Current study
	*Puccinellia banksiensis* Consaul	X			X	New records	Consaul et al. (2008), current study
Potamogetonaceae	*Stuckenia vaginata* (Turcz.) Holub	X					Current study
Amaranthaceae	*Suaeda calceoliformis* (Hook.) Moq.	X					Current study
Brassicaceae	Braya humilis subsp. ellesmerensis J.G. Harris	X					Harris (2006)
	Braya glabella subsp. prostrata J.G. Harris	X					Harris (2006)
	Braya thorild-wulffii subsp. glabrata J.G. Harris	X					Harris (2006)
	*Draba simmonsii* Elven & Al-Shehbaz	X					Elven and Al-Shehbaz (2008)
	*Draba cayouettei* G.A. Mulligan & Al-Shehbaz	X					Al-Shehbaz and Mulligan (2013)
Caryophyllaceae	*Arenaria humifusa* Wahl.		X				Current study
	*Arenaria longipedunculata* Hultén	X			X		Current study
	*Sabulina stricta* (Sw.) Rchb.		X				Current study
Ericaceae	*Andromeda polifolia* L.	X					Current study
	Orthilia secunda subsp. obtusata (Turcz.) Böcher			X			Current study
Fabaceae	Oxytropis deflexa subsp. foliolosa (Hook.) Cody		X				Current study
Lentibulariaceae	*Pinguicula vulgaris* L.		X				Current study
	*Utricularia ochroleuca* R.W. Hartm.	X			X		Current study
Papaveraceae	*Papaver hultenii* Knaben					Confirmed for CAA	Solstad (2009), Elven et al. (2011)
Primulaceae	*Primula egaliksensis* Wormsk.	X					Current study
Ranunculaceae	Coptidium × spitsbergense (Hadač) Luferov & Prob.	X					Current study
Salicaceae	*Salix arctophila* Cockerell ex A. Heller		X				Current study
	*Salix fuscescens* Andersson			X			Current study
Saxifragaceae	*Chrysosplenium rosendahlii* Packer					Confirmed for CAA	Packer (1963), Freeman and Levsen (2007)
	*Saxifraga eschscholtzii* Sternb.				X	New record	Current study
	Saxifraga rivularis subsp. arctolitoralis (Jurtz. & V.V. Petrovsky) M.H. Jørg. & Elven	X					Current study

Three families (Amaranthaceae, Juncaginaceae, Pteridaceae) and seven genera (*Cherleria* L., *Cryptogramma* R.Br., *Platanthera* Rich., *Sabulina* Rchb., *Suaeda* Forssk. ex J.F. Gmel., *Triglochin* L., *Utricularia* L.) are added to the flora. One genus (*Minuartia* L.) is deleted from the flora. We document six recently described taxa as additions to Aiken et al. (2007). Three new species have been described recently from the CAA: *Draba simmonsii* Elven & Al-Shehbaz (Elven and Al-Shehbaz 2008), widely distributed across the CAA, *Draba cayouettei* G.A. Mulligan & Al-Shehbaz from northern Quebec and Southampton Island (Al-Shehbaz and Mulligan 2013) and *Puccinellia banksiensis* Consaul from Banks Island and Arctic coastal Alaska (Consaul et al. 2008; its presence in Nunavut on Victoria Island is documented here). Harris (2006) described three new *Braya* Sternb. & Hoppe subspecies endemic to the CAA: Braya humilis subsp. ellesmerensis J.G. Harris, Braya glabella subsp. prostrata J.G. Harris, and Braya thorild-wulffii subsp. glabrata J.G. Harris. In addition, the first record in Canada and the CAA of a member of the *Puccinellia wrightii* (Scribn. & Merr.) Tzvelev complex was documented by Consaul et al. (2005) on Banks Island, although the precise identity of the single collection remains uncertain pending taxonomic revision of the species complex.

Several taxa are added to the flora of the CAA as a result of recent taxonomic revisions. *Chrysosplenium rosendahlii* Packer, described from Somerset Island (Packer 1963) but subsequently treated as a synonym of *Chrysosplenium tetrandrum* (Scoggan 1978, Aiken et al. 2007), is now considered a distinct species (Freeman and Levsen 2007), a status supported by molecular DNA barcode data (Saarela et al. 2013b). *Papaver hultenii*, described from the Coppermine River on mainland Nunavut and Alaska (Knaben 1959), was considered “apparently common on sandy and gravelly beaches and tundra ridges” on coastal mainland Northwest Territories and north-western mainland Nunavut by Porsild and Cody (1980: 335), but was subsequently treated as a synonym of *Papaver lapponicum* by Kiger and Murray (1997). The species has been confirmed as distinct (Solstad 2009), and as occurring in the western CAA (Elven et al. 2011), where it is now known to be the dominant poppy species on southern Banks and Victoria Islands (collections at CAN and L.J. Gillespie, pers. obs.). *Papaver* sp. “Banks” of southern Banks Island (Solstad 2007, 2009) is now considered conspecific with *Papaver hultenii* (H. Solstad, pers. comm.). The polyphyletic genus *Minuartia* has been divided into eleven genera (Dillenberger and Kadereit 2014), resulting in the addition of two genera, *Cherleria* and *Sabulina*, and the deletion of *Minuartia* from the flora of the CAA.

Several older publications and collections from the Arctic Islands have come to light since the publication of Aiken et al. (2007). While processing older collections at CAN, we became aware of a significant range extension for *Saxifraga eschscholtzii* Sternb., previously known from only one locality in the CAA. Leymus innovatus subsp. velutinus (Bowden) Tzvelev, which was reported for Banks Island in Mason et al. (1972), Porsild and Cody (1980) and Barkworth (2007), was not included in Aiken et al. (2007). Its presence on Banks Island is confirmed here. Additionally, the publication by Thannheiser et al. (2001) with many new distribution records for the Canadian Arctic Islands was overlooked by Aiken et al. (2007). This publication documenting the flora at specific sites on Victoria Island stemmed from fieldwork focusing on plant ecology and phytosociology carried out between 1973 and 1998. No voucher collections were cited in the publication. Collections documenting some of the new records were located at TROM, but others, if they exist, could not be located.

Thannheiser et al. (2001) reported seven species as new to the CAA; of these, three are confirmed here by our new collections (*Andromeda polifolia* L., *Pinguicula vulgaris* L., *Suaeda calceoliformis* (Hokk.) Moq.), two were not new records at the time (*Poa hartzii* Gand., reported earlier in Porsild 1957; *Festuca hyperborea* Holmen, reported in Porsild 1964), and two remain unconfirmed (no voucher specimens found) but are likely not new records. One of these is *Puccinellia deschampsiodes* Th. Sör., a taxon now treated as a synonym of *Puccinellia nuttalliana* (Schult.) Hitchc. (Davis and Consaul 2007), which also includes *Puccinellia borealis* Swallen, previously known from Victoria Island (Porsild 1964, Porsild and Cody 1980). The other is *Parnassia palustris* L., recorded from Johansen Bay; this material has likely been re-identified as *Parnassia kotzebuei* Cham. ex Spreng., which was recorded only from Hadley Bay but with Thannheiser collections present at TROM from both sites. Nine species were considered as new to the western CAA by Thannheiser et al. (2001). Of these, *Sabulina stricta* (Sw.) Rchb. is confirmed here by our new collections and *Carex microglochin* Wahlenb. was confirmed and included in Aiken et al. (2007). Two were not new records: Puccinellia langeana subsp. typica T.J. Sørensen ex Hultén [= Puccinellia tenella subsp. langeana (Berlin) Tzvelev] was reported in Porsild (1964), and *Hedysarum alpinum* L., for which two subspecies were recorded, but only one, Hedysarum alpinum subsp. americanum (Michx. ex Pursh) B. Fedtsch. [= *Hedysarum americanum* (Michx. ex Pursh) Britton], is considered present in the Arctic (Porsild 1957, Porsild and Cody 1980, Elven et al. 2011). *Koenigia islandica* L. and *Eleocharis acicularis* (L.) Roem. & Schult. could not be confirmed since no voucher specimens were located, Potentilla nivea L. subsp. nivea could not be confirmed since it belongs to a taxonomically difficult species complex that has changed over time and largely remains poorly resolved, and two species remain to be confirmed, which also belong to taxonomically difficult species complexes (*Cerastium alpinum* L., *Castilleja caudata* (Pennel) Rebrist.; a specimen of the latter at TROM was determined as Castilleja cf. caudata by R. Elven and I. Alsos).

The majority of the new records described here are assumed to be discoveries of long established species that have simply been overlooked by botanists. One record, *Hordeum jubatum* L., an introduced weedy species found within the Kimmirut town site, is obviously a recent introduction. Documenting the present day flora is essential as baseline data for future studies of floristic changes resulting from the warming climate or from anthropogenic introductions due to increased human traffic.

## Annotated list of new vascular plant records

### MONILOPHYTES

#### Pteridaceae

##### Cryptogramma stelleri

Taxon classificationPlantaePolypodialesPteridaceae

(S.G. Gmel.) Prantl

Fig. 2

###### Common name.

Steller’s rockbrake

###### Distribution.

Disjunct circumboreal (absent from Greenland and Europe)

###### Comments.

This is the first record of the species, genus and family from the CAA and for Nunavut. The genus is easily distinguished from other fern genera in the Arctic Islands by its dimorphic fronds. We discovered one small population on a southeast facing cliff by Fundo Lake on the outskirts of Kimmirut. Plants were small with sterile fronds 3–5(7) cm long and fertile fronds 4–8 cm long, and were growing with moss in horizontal fractures in grey marble.

Uncommon and with a scattered and disjunct distribution, *Cryptogramma stelleri* is found in North America primarily in the western montane boreal and eastern boreal zones (Alverson 1993). It is listed in North America as apparently secure only in Ontario and Quebec, vulnerable to critically imperilled in all other provinces, and vulnerable to possibly extirpated in all states where it occurs and is ranked (NatureServe 2014). Typical habitat in North America is considered to be crevices and rock ledges on calcareous cliffs in boreal habitats (Alverson 1993). Absent from most of the Northwest Territories, Porsild and Cody (1980) recorded it as rare on moist shale slopes in the Richardson and Mackenzie mountains. In northern Quebec it occurs in several small isolated populations mostly in coastal areas near treeline, in cracks on moist shady calcareous cliffs or sometimes on granitic rock in moist, low acid soils on ledges and overhangs (Dignard 2013). Three nearby sites on rocky escarpments near Kangiqsujuaq on the northern Quebec coast occur well within the Arctic (ca. 61°36'N). Our collection from 62°50'44"N on nearby Baffin Island represents a new northern limit for eastern North America. Low spore production, limited dispersal ability, and restricted habitat preference are thought to contribute to its rarity and scattered distribution (Peck et al. 1990, Dignard 2013), and also suggests that this diminutive fern may simply have been overlooked in the past, rather than representing a recent introduction.

###### Specimens examined.

**Canada. Nunavut**: Qikiqtaaluk Region, Baffin Island, Kimmirut, W end of Fundo Lake, ca. 2 km W of hamlet, 62°50'44"N, 69°54'6"W, 40 m, 22 July 2012, *Saarela, Gillespie, Sokoloff & Bull 2774* (ALA, CAN-601315).

### MONOCOTS

#### Cyperaceae

##### Carex bicolor

Taxon classificationPlantaePoalesCyperaceae

Bellardi ex All.

###### Common name.

Two-coloured sedge

###### Distribution.

Circumpolar-alpine

###### Comments.

This is the first report of the species from the western CAA, based on one collection from a sloped sandy riverbank on southern Victoria Island, Nunavut. The species is known from the southeastern CAA (Coats Island, Southampton Island, southern Baffin Island; Porsild and Cody 1980, Aiken et al. 2007). In the western Arctic, *Carex bicolor* is known from adjacent mainland Nunavut (Bathurst Inlet) and Northwest Territories (Porsild and Cody 1980, Saarela et al. 2013a).

###### Specimen examined.

**Canada. Nunavut**: Kitikmeot Region, Victoria Island, W end of Johansen Bay at mouth of Mackenzie Creek, 68°36'4"N, 111°21'7"W, 0–20 m, 20 July 2008, *Gillespie, Saarela, Consaul & Bull 8118* (CAN-592505).

##### Carex brunnescens (Pers.) Poir. subsp. brunnescens

Taxon classificationPlantaePoalesCyperaceae

###### Common name.

Brownish sedge

###### Distribution.

Circumboreal-polar

###### Comments.

This is the first report of the species from the CAA. Our collections were gathered in Katannilik Territorial Park on southern Baffin Island, where the cespitose species was found at three sites in damp, turfy places. It was rare at two sites (only a few scattered plants), and locally common at one site. Associated species include *Betula glandulosa* Michx., Calamagrostis canadensis var. langsdorffii (Link) Inman, *Chamerion angustifolium* (L.) Holub, *Carex arctogena* Harry Sm., *Carex bigelowii* Torr. ex Schwein., *Pedicularis lapponica* L., *Poa arctica* R. Br. and *Taraxacum ceratophorum* (Ledeb.) DC.

This boreal species extends to the treeline across Canada, and into the Arctic zone in northern Quebec and northern Labrador, where it is moderately common (Porsild and Cody 1980, Cayouette 2008), and Greenland (Porsild and Cody 1980). Its discovery on Baffin Island increases the number of *Carex* species known from the CAA to 34. *Carex brunnescens* is classified in Carex sect. Glareosae G. Don (Toivonen 2002); five other species of this section (*Carex ursina* Dewey, *Carex glareosa* Schkuhr ex Wahlenb., *Carex lachenali* Schkuhr, *Carex marina* Dewey) occur in the CAA (Aiken et al. 2007).

###### Specimens examined.

**Canada. Nunavut**: Qikiqtaaluk Region, Baffin Island, Katannilik Territorial Park Reserve, Soper River valley, W bank, near confluence of Willow River, ca. 14 km S of Mount Joy, 63°9'18"N, 69°41'51"W, 41 m, 8 July 2012, *Saarela, Gillespie, Sokoloff & Bull 2232* (CAN-601449); Qikiqtaaluk Region, Baffin Island, Katannilik Territorial Park Reserve, Soper River, W side, S of Livingstone Falls, 63°5'22"N, 69°44'22"W, 67 m, 11 July 2012, *Saarela, Gillespie, Sokoloff & Bull 2346* (ALA, ALTA, CAN-601450, MO, MT, O, UBC, UVIC, WTU); Qikiqtaaluk Region, Baffin Island, Katannilik Territorial Park Reserve, Soper River, 9.5 km S (downstream) of confluence with Livingstone River, W bank, willow stands in gullies at base of E-facing slope, 63°2'32"N, 69°42'47"W, 25 m, 13 July 2012, *Saarela, Gillespie, Sokoloff & Bull 2407* (CAN-601451, MICH, NYBG, WIN).

**Figure 2.**
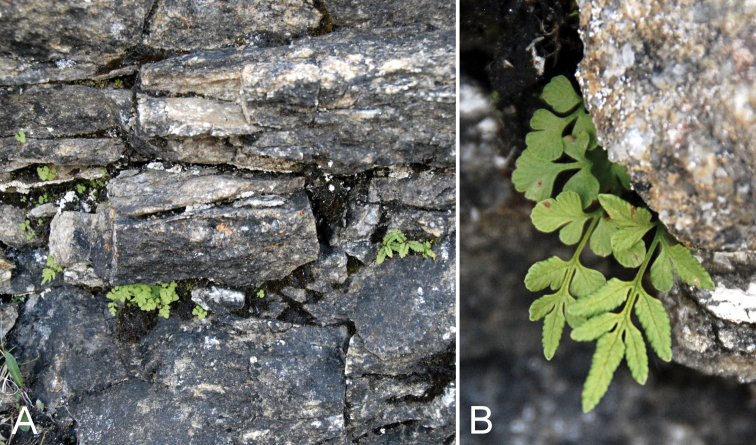
*Cryptogramma stelleri*: **A** habitat **B** habit, *Saarela et al. 2774*. Photographs by L.J. Gillespie.

##### Eriophorum brachyantherum

Taxon classificationPlantaePoalesCyperaceae

Trautv. & C.A. Mey.

Fig. 3

###### Common name.

Short-anther cottongrass

###### Distribution.

Circumboreal-polar

###### Comments.

This is the first report of the species from the western CAA, where we collected it at several sites in the Minto Inlet area of Victoria Island, Northwest Territories. These collections represent a major northeastern range extension of some 350 km from the nearest location on mainland Northwest Territories (Paulatuk; Saarela et al. 2013a) and a north-northeastern extension of some 380 km from the next closest mainland site (Kugluktuk) (Porsild and Cody 1980). It is known from the eastern CAA (one collection on eastern Baffin Island and two on Southampton Island; Porsild and Cody 1980, Aiken et al. 2007). The species was locally common at numerous sites growing in wet sedge meadows, associated with Arctagrostis latifolia (R. Br.) Griseb. subsp. latifolia, *Carex membranacea* Hook., Carex aquatilis var. minor Boott, Carex fuliginosa subsp. misandra (R. Br.) Nyman, *Carex scirpoidea* Michx., *Elymus alaskanus* (Scribn. & Merr.) Á. Löve, *Eriophorum angustifolium* Honck., *Eriophorum triste* (Th. Fr.) Hadač & Á. Löve, Juncus triglumis var. albescens Lange, *Juncus biglumis* L., *Oxyria digyna* (L.) Hill, and *Salix reticulata* L. *Eriophorum brachyantherum* is a cespitose, non-tussock forming species easily distinguished from other cespitose *Eriophorum* species by its tall culms (Ball and Wujek 2002).

###### Specimens examined.

**Canada. Northwest Territories**: Inuvik Region, Victoria Island, 8 km NE of Minto Inlet in valley at small river that feeds into head of inlet, 71°37'9.8"N, 115°26'21.5"W, 100 m, 7 July 2010, *Gillespie, Saarela, Doubt, Bull & Sokoloff 9485* (ALA, CAN-598595, MT, O); Inuvik Region, Victoria Island, N side of small round lake (ca. 1 km diameter), ca. 4 km N of Boot Inlet on N side of Minto Inlet, 71°30'50.8"N, 117°21'43.6"W, 72 m, 11 July 2010, *Gillespie, Saarela, Doubt, Bull & Sokoloff 9673* (ALA, CAN-598605, MT, O); Inuvik Region, Victoria Island, shore E of “Fish Lake” on lower Kuujjua River, 71°12'7.7"N, 116°24'2.7"W, 57 m, 16 July 2010, *Gillespie, Saarela, Doubt, Bull & Sokoloff 9899* (ALA, CAN-598607, MT, O); Inuvik Region, Victoria Island, shore E of “Fish Lake” on lower Kuujjua River, 71°12'7.7"N, 116°24'2.7"W, 57 m, 18 July 2010, *Gillespie, Saarela, Doubt, Bull & Sokoloff 9982* (CAN-598924); Inuvik Region, Victoria Island, valley downstream from the junction of three rivers 6 km NE of head of Minto Inlet, 71°36'31.7"N, 115°27'23"W, 134 m, 21 July 2010, *Gillespie, Saarela, Doubt, Bull & Sokoloff 10091* (CAN-598596); Inuvik Region, Victoria Island, wet sedge meadow on flat to gently sloping plateau E of junction of three rivers 6 km NE of head of Minto Inlet, 71°36'22.8"N, 115°26'30.9"W, 154 m, 21 July 2010, *Gillespie, Saarela, Doubt, Bull & Sokoloff 10102* (ALA, ALTA, ARI, CAN-598910, MT, O, UBC, WIN, US); Inuvik Region, Victoria Island, 8 km NE of Minto Inlet in valley at small river that feeds into head of inlet, 71°37'16.6"N, 115°25'58.7"W, 164 m, 26 July 2010, *Gillespie, Saarela, Doubt, Bull & Sokoloff 10305* (ALA, ARI, CAN-598598, MT, O, UBC, WIN).

#### Juncaceae

##### Luzula wahlenbergii

Taxon classificationPlantaePoalesJuncaceae

Rupr.

###### Common name.

Wahlenberg’s woodrush

###### Distribution.

Circumpolar-alpine

###### Comments.

This is the first collection of this low Arctic species from the western CAA. The taxon is known from several sites on adjacent mainland Nunavut (Porsild and Cody 1980, Cody et al. 1989, Cody 1996, Cody and Reading 2005). Our collection represents a range extension in the central portion of its range of some 330 km north-northwest of the nearest site on mainland Nunavut (George Lake Camp, 65°55'10"N, 107°23'00"W, *Reading 466*, DAO; Cody and Reading 2005). This taxon is now known from eight sites in the CAA: the one reported here, and seven on southeastern Baffin Island. Elsewhere in the Canadian Arctic there are numerous collections of the species from northern Quebec and northwestern North America (Alaska, Yukon, western mainland Northwest Territories) (Porsild and Cody 1980, Swab 2000, Kirschner 2002, Hay 2013).

###### Specimen examined.

**Canada. Nunavut**: Kitikmeot Region, Victoria Island, flat topped steep sided hill, 11 km NE of Johansen Bay airstrip, 68°39'12"N, 110°54'47"W, 120 m, 20 July 2008, *Gillespie, Saarela, Consaul & Bull 8170* (CAN-592326).

**Figure 3.**
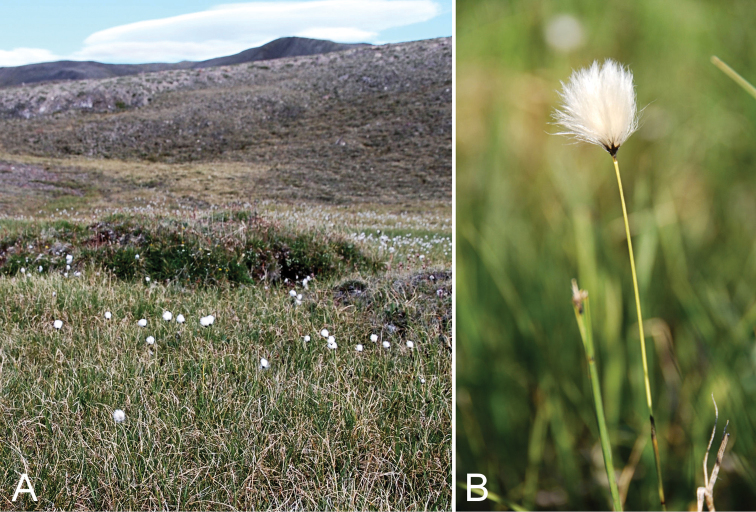
*Eriophorum brachyantherum*: **A** habitat **B** inflorescence, *Saarela et al. 9899*. Photographs by J.M. Saarela.

#### Juncaginaceae

##### Triglochin palustris

Taxon classificationPlantaeAlismatalesJuncaginaceae

L.

Fig. 4

###### Common name.

Marsh arrowgrass

###### Distribution.

Circumboreal-polar

###### Comments.

Discovery of this widely-distributed temperate and facultatively halophytic species growing in wet, brackish habitats at two sites on southern Baffin Island adds a new monocot family, Juncaginaceae, to the flora of the CAA. This taxon is diminutive on Baffin Island, ranging from 6–12 cm tall (larger elsewhere in its range, up to 42.5 cm tall; Haynes and Hellquist 2000a) and therefore easily overlooked, particularly when in flower (fruiting plants are more noticeable). On the mainland, it is known from several Arctic coastal and near-coastal sites in adjacent northern Quebec (Blondeau and Cayouette 2002, Hay 2013) and from a few sites on mainland Nunavut and the Northwest Territories (Porsild and Cody 1980, Blondeau and Cayouette 2002, Saarela et al. 2013a) and southern Greenland (Haynes and Hellquist 2000a). The larger and more robust species *Triglochin maritima* L., which occurs on the mainland Arctic (Porsild and Cody 1980, Hay 2013, Saarela et al. 2013a), is not known from the CAA.

One collection was gathered from a population in wet sandy ground in a dried up depression adjacent to meromictic Soper Lake, associated with *Eriophorum scheuchzeri* Hoppe, *Juncus arcticus* Willd., *Carex bicolor*, and *Dupontia fisheri* R. Br. The second collection was gathered from a sedge meadow at the input of Fundo Lake, associated with *Carex atrofusca* Schkuhr, *Carex gynocrates* Wormsk. ex Drejer, *Carex membranacea* Hook., *Carex microglochin*, *Carex rariflora* (Wahlenb.) Sm., *Carex scirpoidea*, *Eriophorum angustifolium*, *Eriophorum callitrix* Cham., *Eriophorum russeolum* Fr., *Eriophorum scheuchzeri*, *Juncus arcticus*, *Kobresia simpliciuscula* (Wahlenb.) Mack. and *Trichophorum caespitosum* (L.) Hartm.

###### Specimens examined.

**Canada. Nunavut**: Qikiqtaaluk Region, Baffin Island, Katannilik Territorial Park Reserve, Soper Falls, S side of Soper Lake, just SE of Soper Falls, 62°54'1"N, 69°50'54"W, 6 m, 17 July 2012, *Saarela, Gillespie, Sokoloff & Bull 2535* (ALA, CAN-601427, MT); Qikiqtaaluk Region, Baffin Island, Kimmirut, N end of Fundo Lake below Taqaiqsirvik Territorial Park, 62°50'50"N, 69°53'40"W, 35 m, 20 July 2012, *Saarela, Gillespie, Sokoloff & Bull 2652* (CAN-601426, O, WIN).

**Figure 4.**
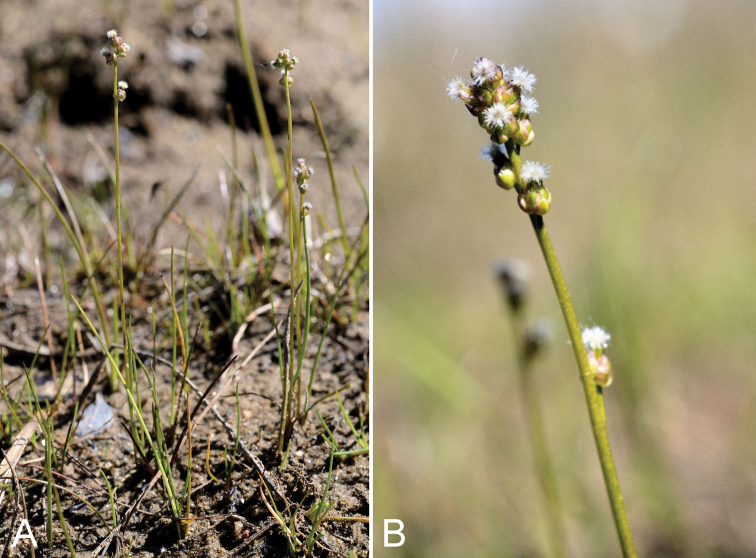
*Triglochin palustris*: **A** habit **B** inflorescence, *Saarela et al. 2535*. Photographs by R.D. Bull.

#### Orchidaceae

##### Corallorhiza trifida

Taxon classificationPlantaeAsparagalesOrchidaceae

Chatelain

Fig. 5

###### Common names.

Northern coralroot, early coralroot

###### Distribution.

Circumboreal-polar

###### Comments.

Our collections represent the first record of the species in the western CAA, and the second for the eastern CAA and Baffin Island. Thought to be the only orchid in the CAA (Aiken et al. 2007, but see *Platanthera obtusata*), it was previously known from only one collection and two sites in Auyuittuq National Park, Baffin Island (Gould 1997). Common throughout boreal Canada, its range is scattered and sparse north of the tree-line to the mainland Arctic coast from the Yukon to Bathurst Inlet, Nunavut, and along the Hudson Bay coast (Porsild and Cody 1980). In their treatment of *Corallorhiza trifida* for the Flora of North America, Magrath and Freudenstein (2002) reported the species from the western CAA. They mapped two dots on Victoria Island: one centered on the Cambridge Bay area, the other on south-central Victoria Island; and they shaded the southern half of Prince of Wales Island. We are not aware of specimens or other literature reports for these records; they do not appear in Freudenstein’s (1997) revision of *Corallorhiza* in North America, nor does J. Freudenstein (pers. comm. 2014) know the source of these records (L. Magrath, first author of the FNA treatment, is deceased). Our collection from south-central Victoria Island (incidentally, this is one of the same areas mapped in Magrath and Freudenstein 2002) is the only confirmed record for the western CAA. It was recorded as uncommon on the low, densely vegetated, south-facing bank of a creek near its mouth, on a mostly sandy substrate, with *Dryas integrifolia*, *Bistorta vivipara* (L.) Gray, and Hedysarum boreale subsp. mackenziei (Richardson) S.L. Welsh (*Salix* and *Arctous rubra* (Rehder & E.H. Wilson) Nakai nearby).

In the Soper River valley on southern Baffin Island we found the species to be scattered, but never common, on densely vegetated river flats, riverbanks, and peaty wet meadows at several localities. Our three collections increase the number of records for Baffin Island to four. In adjacent northern Quebec, the species occurs along the coast and in the interior, known from only three Arctic localities (Houle 2013).

**Figure 5.**
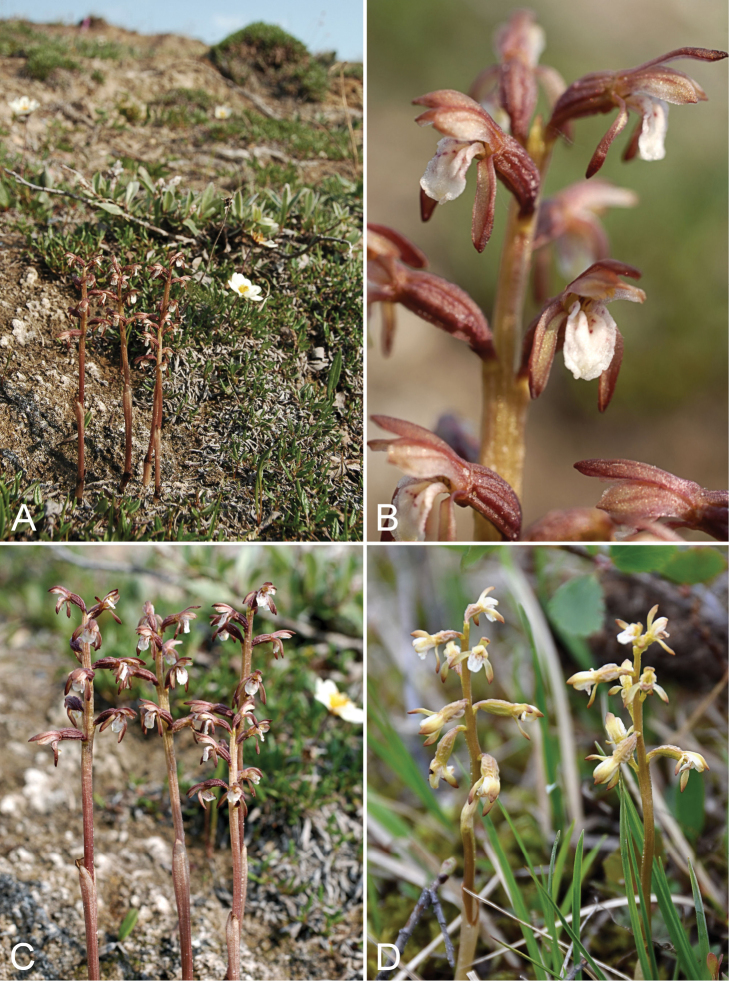
*Corallorhiza trifida*: **A** habitat **B** inflorescence **C** habit, *Gillespie et al. 8093* **D** habit, *Saarela et al. 1970*. Photographs by R.D. Bull.

The species is a near-complete mycoheterotroph (Zimmer et al. 2008, Cameron et al. 2009), and in most of its range plants are green to yellow-green in colour (e.g., see photo in Houle 2013: 322). Freudenstein (1997) noted that lighter-coloured individuals tend to occur in more southern, forested areas, whereas darker-coloured forms occur in exposed northern sites, such as tundra. Earlier observations of the species at its northern limits in Canada are consistent with this (Gould 1997, Saarela et al. 2013a: Fig. 19). Our collection from Victoria Island was prominently reddish-brown throughout (anthocyanic) (Fig. 5A–C); those from the Soper River valley less so (Fig. 5D). None of the populations we observed was as large as a population of 56 individuals found in Auyuittuq National Park of Canada, Baffin Island (Gould 1997). One population collected and surveyed in the Soper River valley had 19 stems in a 5 × 3 m area (*Saarela et al. 1970*); the population collected on Victoria Island had 14 stems in two clumps (*Gillespie et al. 8093*). A fourth occurrence was observed but not collected in the Soper River valley (near confluence of Willow River, ca. 14 km S of Mount Joy, 63°9'24"N, 69°41'35"W).

###### Specimens examined.

**Canada. Nunavut**: Kitikmeot Region, Victoria Island, W end of Johansen Bay at mouth of Mackenzie Creek, 68°36'4"N, 111°21'7"W, 0–20 m, 20 July 2008, *Gillespie, Saarela, Consaul & Bull 8093* (CAN-592381; Qikiqtaaluk Region, Baffin Island, Katannilik Territorial Park Reserve, densely vegetated river flat near Mount Joy, ca. 5 m wide band between river and dry stony floodplain, 63°14'52.7"N, 69°36'45.7"W, 75 m, 1 July 2012, *Saarela, Gillespie, Sokoloff & Bull 1970* (CAN-601648); Qikiqtaaluk Region, Baffin Island, Katannilik Territorial Park Reserve, peaty wet meadow along Soper River, ca. 0.5 km N of Mount Joy, 63°15'3"N, 69°36'6"W, 86 m, 2 July 2012, *Saarela, Gillespie, Sokoloff & Bull 2036* (CAN-601649); Qikiqtaaluk Region, Baffin Island, Katannilik Territorial Park Reserve, Soper River, high water mark along riverbank, ca. 13 km downstream (S) of its confluence with the Livingstone River, 62°59'40"N, 69°42'46"W, 35 m, 13 July 2012, *Saarela, Gillespie, Sokoloff & Bull 2415* (CAN-601650).

##### Platanthera obtusata (Banks ex Pursh) Lindl. subsp. obtusata

Taxon classificationPlantaeAsparagalesOrchidaceae

Fig. 6

Habenaria obtusata (Banks ex Pursh) Richards
Lysiella obtusata (Banks ex Pursh) Rydb.

###### Common name.

Northern bog orchid

###### Distribution.

Boreal North America

###### Comments.

This is the first record for this genus and species, and the second species of orchid discovered (see *Corallorhiza trifida*), in the CAA (Aiken et al. 2007). The species is currently considered to include two subspecies; all North American plants belong to subsp. obtusata, while Eurasian plants are treated as subsp. oligantha (Turcz.) Hultén (Sheviak 2003, Elven et al. 2011). This wide-ranging boreal species of damp or wet, turfy places (Correll 1978) is also found beyond the treeline in Canada from northern Yukon to northern Quebec (Porsild and Cody 1980, Cody 2000, Cody et al. 2003, Sheviak 2003, Saarela et al. 2013a). Porsild (1955) suggested that the species is likely to be found in southern areas of the western Arctic Islands, but it has not yet been found there. In Arctic Quebec, the species has been reported as occurring along the east coast of Hudson Bay (Polunin 1940, Porsild and Cody 1980, Sheviak 2003, Houle 2013) and at five sites on the north-central Ungava Peninsula (Maycock and Matthews 1966, Blondeau and Cayouette 2002, Houle 2013). Blondeau and Cayouette (2002) reported the species from two sites near Douglas Harbour along the northern coast, just south of Kimmirut, Baffin Island (mapped in Houle 2013). At one site the species was uncommon at the base of a scree slope along a stream margin, and at the second only a few individuals were found growing among rocks.

Along the Soper River on southern Baffin Island we collected three populations. The first (*Saarela et al. 2197*) had two subpopulations with a total of 80 plants, the second (*Saarela et al. 2209*) came from a population of over 100 plants in a 10 m^2^ area, and the third (*Saarela et al. 2488*) was from a population of over 250 plants in a 80 m^2^ area. Near the third population was an even larger population estimated at over 1000 plants that was not collected. These populations were found in moist sedge-willow hummocks set on small hills and valleys on the lower slopes of the Soper Valley away from the banks of the Soper River, growing in association with *Betula glandulosa*, *Salix arctophila* Cockerell ex A. Heller, *Salix calcicola* Fernald & Wiegand, *Salix reticulata*, *Empetrum nigrum* L., *Rhododendron lapponicum* (L.) Wahlenb., *Equisetum arvense* L., *Cassiope tetragona* (L.) D. Don, *Vaccinium uliginosum* L., and *Vaccinium vitis-idaea* L. While the first two populations were encountered within a few kilometers of each other, we encountered the third, largest population 20 kilometers away, suggesting that other populations may occur in the area where habitat is suitable.

###### Specimens examined.

**Canada. Nunavut**: Qikiqtaaluk Region, Baffin Island, Katannilik Territorial Park Reserve, Soper River valley, W bank, ca. 12 km S of Mount Joy, meadow along river opposite Group/Warden Cabin #7, 63°9'50"N, 69°40'2"W, 40 m, 8 July 2012, *Saarela, Gillespie, Sokoloff & Bull 2197* (CAN-601651); Qikiqtaaluk Region, Baffin Island, Katannilik Territorial Park Reserve, Soper River valley, W bank, ca. 1 km S of Mount Joy, moderate S-facing slope, 63°9'39"N, 69°40'29"W, 55 m, 8 July 2012, *Saarela, Gillespie, Sokoloff & Bull 2209* (CAN-601276); Qikiqtaaluk Region, Baffin Island, Katannilik Territorial Park Reserve, Soper River, 18.5 km downstream (S) of its confluence with the Livingstone River, 2 km S of Emergency Cabin #8, W side of river, 62°59'28"N, 69°43'30"W, 67 m, 15 July 2012, *Saarela, Gillespie, Sokoloff & Bull 2488* (ALA, CAN-601652, O).

**Figure 6.**
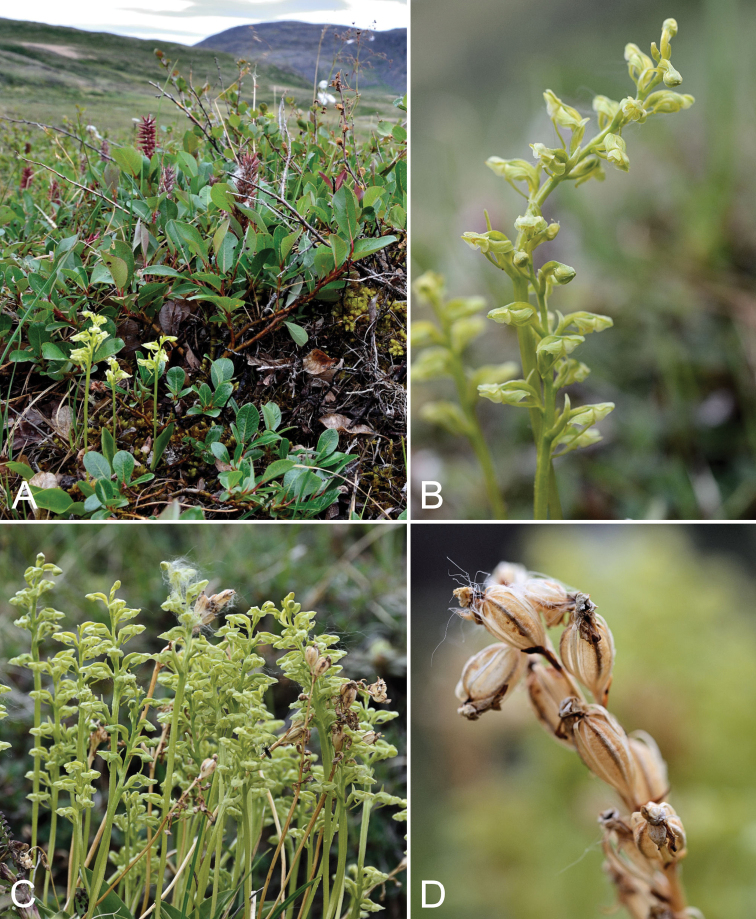
Platanthera obtusata subsp. obtusata: **A** habitat, *Saarela et al. 2197* **B** inflorescence **C** habit **D** old fruits, *Saarela et al. 2209*. Photographs by R.D. Bull.

#### Poaceae

##### Calamagrostis stricta subsp. groenlandica

Taxon classificationPlantaePoalesPoaceae

(Schrank) Á. Löve

Fig. 7

###### Common name.

Slim-stemmed reedgrass

###### Distribution.

Circumboreal-polar

###### Comments.

Our new collections confirm the presence of this taxon in the eastern CAA. We collected specimens from several populations in Katannilik Territorial Park on southern Baffin Island, in mesic to wet tundra habitats. The species is documented in the western CAA (Banks Island, Melville Island, Prince Patrick Island; Aiken et al. 2007, as Calamagrostis neglecta subsp. groenlandica (Schrank) Matuszk). Porsild and Cody (1980) reported the taxon (as *Calamagrostis neglecta* (Ehrh.) G. Gaertn., B. Mey. & Scherb.) from Devon Island, the Cumberland Peninsula of Baffin Island and Coats Island, but these records were not mapped in Aiken et al. (2007), nor could specimens be located at CAN or DAO. Associated species on Baffin Island include *Agrostis mertensii* Trin., Arctagrostis latifolia subsp. latifolia, *Betula glandulosa*, *Carex rariflora*, *Carex membranacea*, *Empetrum nigrum*, *Eriophorum vaginatum*, *Huperzia selago* (L.) Bernh. ex Schrank & Mart., *Luzula wahlenbergii*, Rhododendron tomentosum Harmaja subsp. decumbens (Aiton) Elven & D.F. Murray, *Salix arctica* Pall., *Salix arctophila*, and *Vaccinium vitis-idaea*.

###### Specimens examined.

**Canada. Nunavut**: Qikiqtaaluk Region, Baffin Island, Katannilik Territorial Park Reserve, Soper River valley, E bank, large sedge meadow with several small ponds ca. 12.5 km S of Mount Joy, 0.5 km S of Group/Warden Cabin #7, 63°9'35"N, 69°40'3"W, 41 m, 7 July 2012, *Saarela, Gillespie, Sokoloff & Bull 2191* (ALTA, CAN-601348, MO, US); Qikiqtaaluk Region, Baffin Island, Katannilik Territorial Park Reserve, Soper River, E bank, 12 km S of Mount Joy along river, at Group/Warden Cabin #7, 63°9'44"N, 69°39'28"W, 50 m, 9 July 2012, *Saarela, Gillespie, Sokoloff & Bull 2255* (ALA, CAN-601345); Qikiqtaaluk Region, Baffin Island, Katannilik Territorial Park Reserve, Soper River, 5 km S (downstream) of confluence with Livingstone River, E bank, 63°4'32"N, 69°42'11"W, 30 m, 13 July 2012, *Saarela, Gillespie, Sokoloff & Bull 2398* (CAN-601347); Qikiqtaaluk Region, Baffin Island, Katannilik Territorial Park Reserve, Soper River, 18.5 km downstream (S) of its confluence with the Livingstone River, 2 km S of Emergency Cabin #8, E bank of river, 62°59'13"N, 69°42'48"W, 28 m, 14 July 2012, *Saarela, Gillespie, Sokoloff & Bull 2442* (ALTA, CAN-601346); Qikiqtaaluk Region, Baffin Island, Katannilik Territorial Park Reserve, Soper Falls/Soper Lake, S side of Soper River, 62°54'6"N, 69°51'2"W, 8 m, 18 July 2012, *Saarela, Gillespie, Sokoloff & Bull 2576* (CAN-601344, O).

**Figure 7.**
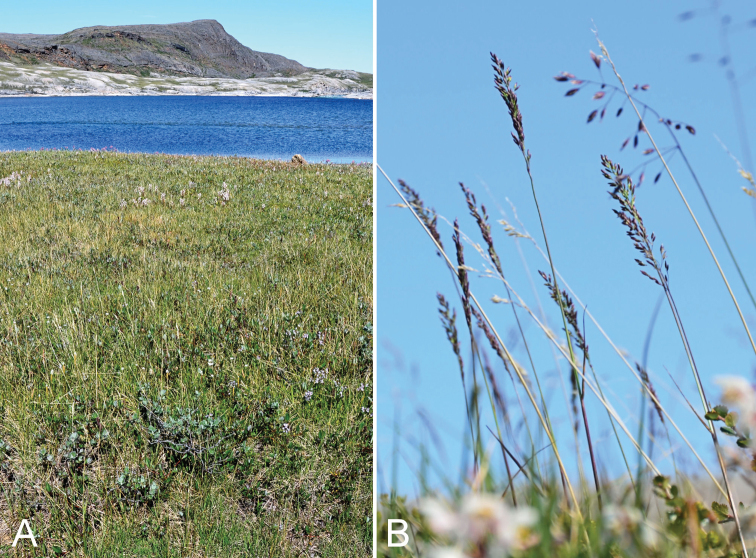
Calamagrostis stricta subsp. groenlandica: **A** habitat **B** habit, *Saarela et al. 2576*. Photographs by R.D. Bull.

##### Hordeum jubatum L. subsp. jubatum

Taxon classificationPlantaePoalesPoaceae

Fig. 8

###### Common name.

Foxtail barley

###### Distribution.

North America–NE Asia

###### Comments.

*Hordeum jubatum* is a widely distributed species that grows in meadows, along rivers, around lakes, and in disturbed habitats such as roadsides (von Bothmer et al. 2007). Two subspecies are recognized: subspecies *jubatum* and *intermedium* Bowden, which differ in the lengths of their glumes and lemma awns of the central spikelets (Bowden 1962, von Bothmer et al. 2007); the variation in these characters is continuous and some intermediate specimens cannot be assigned to subspecies (Bowden 1962, Baden and von Bothmer 1994). Bowden (1962) considered subsp. intermedium to be a hybrid between *Hordeum jubatum* s.s. and *Hordeum brachyantherum* Nevski, but to our knowledge this hypothesis has not been tested with molecular data. Some authors treat subsp. intermedium as a separate species, *Hordeum caespitosum* Scribn. (e.g., Baum and Bailey 1994). Hordeum jubatum subsp. jubatum is a weedy species native from eastern Siberia and northeastern China through North America to Mexico, and it is introduced to South America, Europe and Central Asia (Baden and von Bothmer 1994, von Bothmer et al. 2007). It is generally considered to be native in western North America and adventive in eastern and southeastern North America (e.g., Hitchcock 1951, von Bothmer et al. 2007), but some authors consider it native across North America (Baden and von Bothmer 1994). Bowden (1962) noted the subspecies to be expanding its range in northern Canada. Hordeum jubatum subsp. intermedium grows in central and western Canada and United States, the Magdalene Islands, Quebec, and is disjunct in southern Mexico (Bowden 1962, Baden and von Bothmer 1994, von Bothmer et al. 2007).

Although the species is distributed primarily in temperate and sub-Arctic regions of North America there are sporadic collections of both subspecies from Arctic regions of Alaska (Klein 2011, Skinner et al. 2012) and Canada. On Canada’s mainland Arctic, Hordeum jubatum subsp. intermedium has been recorded from Hood River, Nunavut (*Anderson 473* in 1915, CAN-39857 & CAN-514373; Macoun and Holm 1921, Bowden 1962) and from Tuktoyaktuk, Northwest Territories (*Aiken & McLachlan 87-221* in 1987, CAN-530893). Two records of *Hordeum jubatum* s.l. from Ungava Bay in northern (Arctic) Quebec and one from western Greenland are mapped in von Bothmer et al. (2007).

*Hordeum jubatum* was apparently accidentally introduced as early as the 1960s to Apex (near Iqaluit, Baffin Island, CAA) with straw used as animal feed and/or packing material (Aiken et al. 2007). Plants were observed (and collected) in the same area (around the Hudson’s Bay Company house) in the mid- to late-1980s (*Aiken, Campbell & Robinson 86-445* in 1986, CAN-518325; *Aiken, Campbell & Robinson 86-337* in 1986, CAN-518217; *Aiken 89-115* in 1989, CAN-541784). These three specimens were not previously determined to subspecies. The two 1986 collections are intermediate between subspecies *jubatum* and *intermedium* and the 1989 collection is subsp. jubatum. It is unknown if these collections represent the same or separate introductions. The species was observed in the same area in 1998 and 2002 (no collections were made), but the site was overgrown by willows in 2005 and the species was absent (Aiken et al. 2007). We were at the site in July 2012 and did not encounter the species. There is also a 2003 collection from a separate locality in nearby Iqaluit (across from Joamie Ilinniarvik School, *Mallory s. n.*, CAN-585777). The label on this specimen indicates “possibly an accidental introduction as part of earlier project to hydro-seed grass around the school.” It is not known if the species persists in the Iqaluit area.

**Figure 8.**
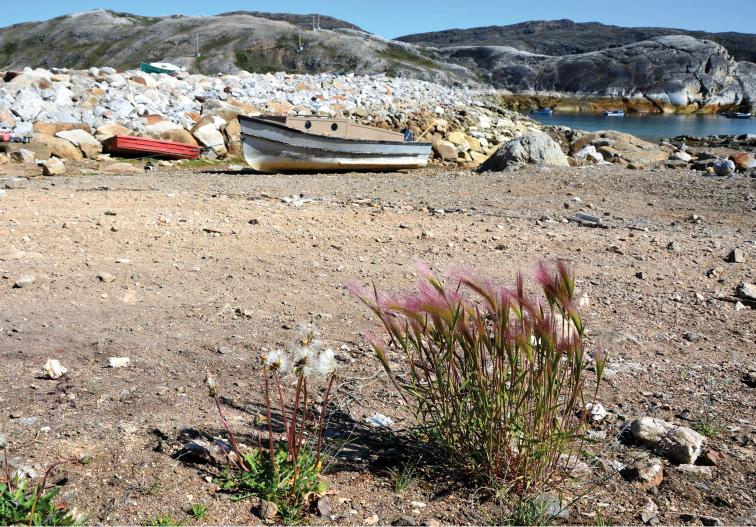
Hordeum jubatum subsp. jubatum: habitat, *Saarela et al. 2737*. Photograph by R.D. Bull.

We found three robust plants of Hordeum jubatum subsp. jubatum in the community of Kimmirut in 2012, adding a second area of occurrence for the species on Baffin Island. Two plants were growing in a lush sewage runoff area near the garbage dump on slopes well above the coastal high tide line with *Chamerion latifolium* (L.) Holub, *Poa alpina* L., *Poa glauca* Vahl, *Salix glauca* L., *Stellaria longipes* Goldie and *Taraxacum lapponicum* Kihlm. ex Hand.-Mazz., and one in the hamlet, growing on a rocky, sandy beach adjacent to the coast associated with *Poa arctica* and *Taraxacum lapponicum* (Fig. 8). Based on the few individuals found in Kimmirut, these likely represent very recent introductions, which may have arrived naturally (dispersal by birds, for example) or been introduced unintentionally by humans. The presence of this species in Kimmirut should be monitored to determine if it is increasing its presence there, particularly at the sewage runoff site where a high nutrient load supports lush plant growth (J.M. Saarela and P.C. Sokoloff, pers. obs.).

###### Specimens examined.

**Canada. Nunavut**: Qikiqtaaluk Region, Baffin Island, Kimmirut, rocky sandy slope between Northern Store and coast, 62°50'57"N, 69°52'12"W, 68 m, 22 July 2012, *Saarela, Gillespie, Sokoloff & Bull 2737* (ALA, ALTA, CAN-601368); Qikiqtaaluk Region, Baffin Island, Kimmirut, S end of hamlet, below garbage dump and above high tide line at coast, 62°50'26"N, 69°52'20"W, 68 m, 22 July 2012, *Saarela, Gillespie, Sokoloff & Bull 2755* (CAN-601369, O, US).

##### Leymus innovatus subsp. velutinus

Taxon classificationPlantaePoalesPoaceae

(Bowden) Tzvelev

###### Common Name.

Northern downy ryegrass

###### Distribution.

American Beringia

###### Comments.

Although not reported in Aiken et al. (2007) for the CAA, this species was first reported for the CAA from Banks Island by Mason et al. (1972, as *Elymus innovatus* Beal), based on a collection from the Masik River Valley (71°37'N, 123°6'W, 20 July 1968, *W.R.M. Mason 93*, DAO-543555, not seen). There is also a collection in CAN (two sheets) from Sachs Harbour (Banks Island, Northwest Territories), previously determined as *Agropyron violaceum* (Hornem.) Lange (det. A.E. Porsild) and Elymus alaskanus subsp. latiglumis (Scribn. & J.G. Smith) Á. Löve (det. M.E. Barkworth, 1993), that has been re-determined as this species (det. J.M. Saarela). The taxon was mapped on southern Banks Island by Porsild and Cody (1980) and Barkworth (2007), probably based on the Mason collection and/or one or more correctly-determined duplicates of the Sachs Harbour collection in other herbaria. It grows in Alaska, the Yukon Territory, and the western Northwest Territories (Barkworth 2007) with Sachs Harbour and the Masik River Valley the only known locations in the CAA. *Leymus innovatus* (Beal) Pilg. and *Leymus mollis*—the only two species of the genus in the CAA—may be distinguished by the following key (adapted from Barkworth 2007):

**Table:** 

1	Lemmas unawned, 11–20 mm long; glumes tapering from midlength or above, flat or rounded on the back, apices acute	***Leymus mollis***
–	Lemmas awned, 7–12 mm long; glumes tapering from the base to the nearly subulate apices	***Leymus innovatus***

###### Specimens examined.

**Canada. Northwest Territories**: Banks Island, Sachs Harbour, 71°58'N, 125°15'W, 17–25 July 1969, *M. Kuc 405* (CAN-432022, CAN-432023).

##### Leymus mollis (Trin.) Pilg. subsp. mollis

Taxon classificationPlantaePoalesPoaceae

###### Common name.

Sea lyme-grass, American dune grass

###### Distribution.

Amphi-Pacific–North America

###### Comments.

Two subspecies of *Leymus mollis* are recognized in North America: subsp. mollis and subsp. villosissimus (Scribn.) Á. Löve & D. Löve (Bowden 1957, Barkworth 2007, Elven et al. 2011). Leymus mollis subsp. villosissimus is an Arctic taxon, distributed from Siberia to Greenland, and common in the low CAA, while subsp. mollis grows along the east and west coasts of North America, along the Arctic coast of Quebec, in some interior locations (Great Slave Lake, for example) and in Greenland (Bowden 1957, Aiken et al. 2007, Barkworth 2007). Subspecies *mollis* has not previously been reported from the CAA (Bowden 1957, Aiken et al. 2007) and our collection from southern Baffin Island is thus the first record for the region. The collection was made on the outer sandy floodplains of Soper Lake, where the species was uncommon; subspecies *villosissimus* was more common in the region.

###### Specimens examined.

**Canada. Nunavut**: Qikiqtaaluk Region, Baffin Island, Katannilik Territorial Park Reserve, Soper Falls, south side of Soper Lake, just southeast of Soper Falls, 17 July 2012, 62°54”08'N, 69°50”42'W, 6 m, *Saarela, Gillespie, Sokoloff & Bull 2529* (CAN-601371).

##### Puccinellia banksiensis

Taxon classificationPlantaePoalesPoaceae

Consaul

Fig. 9

###### Common name.

Dwarf alkaligrass

###### Distribution.

Arctic NW North America

###### Comments.

This species was described recently from three localities on southern Banks Island, Northwest Territories, and one locality in northern Alaska (Consaul et al. 2008). Saarela et al. (2013a) reported two collections from the lower Brock River on mainland Northwest Territories. Here we report six new localities for the species from southwestern Victoria Island—the first records for this island and for Nunavut, expanding the species’ range eastwards in the CAA.

###### Specimens examined.

**Canada. Nunavut**: Victoria Island, Oterkvik Point vicinity, ca. 9 km N of Coronation Gulf coast, 12 km N of point, 68°35'34"N, 112°35'43"W, 40–50 m, 5 July 2012, *Gillespie, Saarela, Consaul & Bull 7549* (CAN-600906); Johansen Bay, 18 km east-northeast of airstrip, Nakoyoktok River at outflow of large unnamed lake, 18 July 2008, 68°39'25"N, 110°42'30"W, 20–30 m, *Gillespie, Saarela, Consaul & Bull 8055* (CAN-592678), *8055-2* (CAN-592239); Victoria Island, Johansen Bay, main air landing strip, 20 July 2008, 68°35'50"N, 111°06'59"W, 120 m, *Gillespie, Saarela, Consaul & Bull 8077* (CAN-592679); Victoria Island, pingo, 23 km west of Johansen Bay airstrip, 20 July 2008, 68°36'23"N, 111°40'22"W, 100–120 m, *Gillespie, Saarela, Consaul & Bull 8146-2* (CAN-592688); Victoria Island, tundra between Sinclair Creek North Warning System site (abandoned DEW-line site) and coast, 68°44'35"N, 109°06'15"W, 20–70 m, 22 July 2008, *Gillespie, Saarela, Consaul & Bull 8240* (CAN-592705); Victoria Island, south of Sinclair Creek North Warning System site (abandoned DEW-line site), approximately 1 km N of coast, 22 July 2008, 68°43'14"N, 109°05'10"W, 10–20 m, *Gillespie, Saarela, Consaul & Bull 8261* (ALA, CAN-592689, MT, O); Victoria Island, disturbed ground in the vicinity of the Sinclair Creek North Warning System site (abandoned DEW-line site), 68°45'5"N, 109°06'20"W, 75 m, 23 July 2008, *Gillespie, Saarela, Consaul & Bull 8339* (ALA, CAN-592707, MT, O, US).

**Figure 9.**
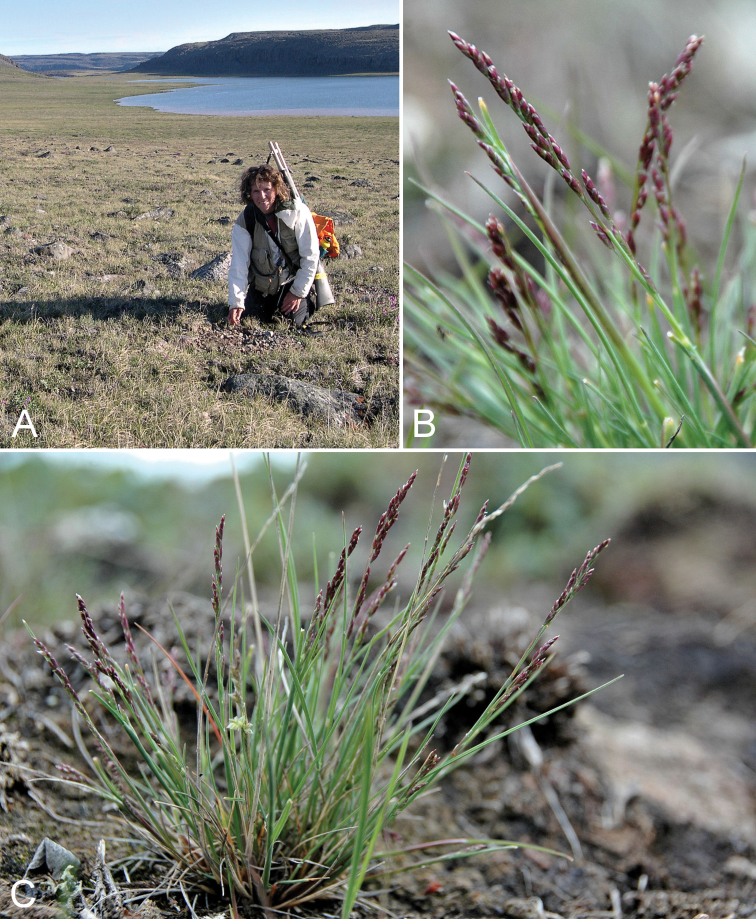
*Puccinellia banksiensis*: **A** habitat, with Laurie Consaul who described the species **B** inflorescence **C** habit, *Gillespie et al 8055*. Photographs by L.J. Gillespie (**A**), R.D. Bull (**B, C**).

#### Potamogetonaceae

##### Stuckenia vaginata

Taxon classificationPlantaeAlismatalesPotamogetonaceae

(Turcz.) Holub

Fig. 10

Potamogeton vaginatus Turcz.
Stuckenia subretusa (Hagstr.) Holub

###### Common name.

Big-sheathed pondweed

###### Distribution.

Circumboreal

###### Comments.

This collection is the first record of this primarily boreal species for the CAA. The species has a scattered distribution across Canada north to treeline and reaches the Arctic in coastal Yukon, coastal mainland Northwest Territories, and southeastern mainland Nunavut (Porsild and Cody 1980, Haynes and Hellquist 2000b, Saarela et al. 2013a). The nearest site on the mainland is in the Northwest Territories near the coast just northwest of the border with Nunavut (*Scotter & Zoltai 90-494*, DAO; Saarela et al. 2013a), some 440 km west-northwest of our site. A slightly closer record (ca. 400 km) was mapped from eastern Great Bear Lake in Porsild and Cody (1980) (presumably based on a specimen collected by A.E. Porsild housed at GH, as cited by Raup 1947, no collection number given). A probable duplicate at CAN (Great Bear Lake, N shore of McTavish Arm, Black Rock, Laurentian, about 66°20'N, 118°30'W, 6 August 1928, *Porsild & Porsild 6186*, CAN-7215, det. *Potamogeton vaginatus* by M. Fernald) was re-determined as Coleogeton filiformis subsp. occidentalis (J.W. Robbins) Les & R.R. Haynes (= Stuckenia filiformis subsp. occidentalis (J.W. Robbins) R.R. Haynes, Les & M. Král) by C.B. Hellquist, and the site was not mapped for *Stuckenia vaginata* in Haynes and Hellquist (2000b).

Following the treatment by Kaplan (2008) *Stuckenia vaginata* may be distinguished by its open leaf sheaths from *Stuckenia filiformis* (Pers.) Börner, the only species of the family known to occur in the CAA prior to this collection. Although *Stuckenia vaginata* is generally more robust in habit with wider leaf sheaths and more numerous whorls of flowers on the inflorescence (usually 7–9 versus 3–6 in *Stuckenia filiformis*), our collection from the northern edge of its range was somewhat intermediate in size with few young inflorescences (and no fruit) having 5–7 whorls of flowers.

The taxonomy of *Stuckenia* Borner is complex and there are several conflicting taxonomic treatments (e.g., Tolmachev et al. 1995, Haynes and Hellquist 2000b, Kaplan 2008; see discussion in Elven et al. 2011). Our collection was initially identified by R. Elven in 2009 as *Stuckenia subretusa* (Hagstr.) Holub, a primarily Russian Arctic species, based on its retuse or subretuse leaf apices. Although included in the Panarctic Flora, Elven et al. (2011) were not fully convinced that it should be treated as distinct and suggested a possible alternative treatment within a variable *Stuckenia filiformis*. Tolmachev et al. (1995) recognized *Stuckenia subretusa* in their treatment for the Russian Arctic, but suggested it might be an arctic race of *Stuckenia vaginata*. Kaplan (2008) in his revision of Asian *Stuckenia* treated *Stuckenia subretusa* as a synonym of *Stuckenia vaginata* (both have open leaf sheaths contrasting with the fused leaf sheaths of *Stuckenia filiformis*); he found leaf apex shape to vary within specimens and (sub)retuse leaf apices on collections from across the range of *Stuckenia vaginata*. Saarela et al. (2013b) in their barcode study of Canadian Arctic Island vascular plant species found that the *rbcL* and *matK* sequences of our collection (as *Stuckenia subretusa*) were identical to those of *Stuckenia vaginata*, and different from *Stuckenia filiformis*, consistent with Kaplan’s (2008) treatment. Here we follow Kaplan (2008) in treating *Stuckenia subretusa* as a synonym of *Stuckenia vaginata*, but also recognize that the species complex in North America is in need of further study. If *Stuckenia subretusa* is considered a distinct species, our collection would represent the first record for Canada (and is the one referred to in Elven et al. (2011) documenting presence of the species on Victoria Island and in Canada). If treated within *Stuckenia filiformis*, our collection would represent the first record for the western CAA.

###### Specimens examined.

**Canada. Nunavut**: Victoria Island, Kitikmeot Region, Johansen Bay, 18 km ENE of airstrip, Nakoyoktok River at outflow of large unnamed lake, 68°39'25"N, 110°42'30"W, 20–30 m, 18 July 2008, *Gillespie, Saarela, Consaul & Bull 8048* (ALA, ALTA, BABY, CAN-592375, MT, O, UBC, US).

**Figure 10.**
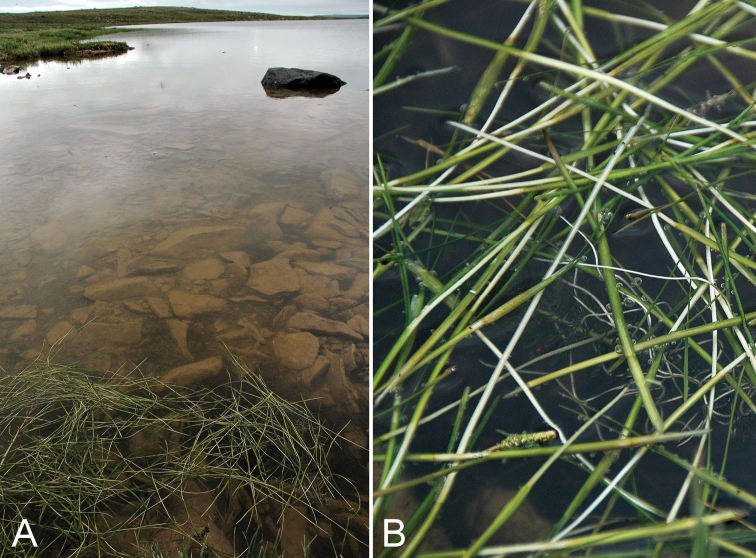
*Stuckenia vaginata*: **A** habitat **B** habit, *Gillespie et al. 8048*. Photographs by R.D. Bull.

### EUDICOTS

#### Amaranthaceae

##### Suaeda calceoliformis

Taxon classificationPlantaeCaryophyllalesAmaranthaceae

(Hook.) Moq.

Fig. 11

###### Common name.

Horned sea-blite

###### Distribution.

North America

###### Comments.

This species was first recorded as occurring at Johansen Bay along southern Victoria Island by Thannheiser et al. (2001; voucher at TROM, not seen); however, the species was not included in Aiken et al. (2007) and we thus report it here. We collected *Suaeda calceoliformis* at five sites on Victoria Island: three on southern Victoria Island (Nunavut), and two in the vicinity of Minto Inlet on north-western Victoria Island (Northwest Territories). These are the first records for this family, genus and species in the CAA. We initially mis-identified our collections as the annual *Koenigia islandica* (Polygonaceae), a superficially similar species known from the adjacent mainland and the eastern Arctic Islands (Aiken et al. 2007, Porsild and Cody 1980). The true identity of our material was revealed upon collection of DNA barcode data (Saarela et al. 2013b), which placed them with other *Suaeda* individuals and distinct from *Koenigia*. Re-examination of the very small specimens confirmed their identity as *Suaeda calceoliformis*.

This species is found in saline and disturbed environments in the western and midwestern United States north to south-western Yukon, along southern James Bay and coastal areas of eastern Canada and north-eastern United States (Bassett and Compton 1978, Cody 2000, Riley 2003, Ferren Jr. and Schenk 2004). It is also known from one sub-Arctic site on the northern side of Great Bear Lake and four areas in the western mainland Arctic: Tuktoyaktuk Peninsula; Rae River mouth, Kugluktuk area; Walker Bay, Kent Peninsula; and Paulatuk and Lower Brock Lagoon (Bassett and Compton 1978, Cody et al. 2003, Ferren Jr. and Schenk 2004, Porsild and Cody 1980; specimen citations given in Saarela et al. 2013a). The species was treated as a rare plant for the Canadian Arctic (McJannet et al. 1993). Our five collections from Victoria Island double the number of known sites for this species in the Canadian Arctic. It has probably been overlooked by collectors in its Arctic range, as it is very small and has fairly specialized habitat requirements.

*Suaeda calceoliformis* displays a wide degree of phenotypic plasticity throughout its range; for example, its height ranges from 5 cm to 1 m in continental Canada (Ferren Jr. and Schenk 2004). Our collections range from 1–4 cm, with the smallest plants often only possessing a single inflorescence. Habitats on Victoria Island include saline depressions inland and coastal saline flats, and the species was typically found growing in association with *Puccinellia arctica* (Hook.) Fernald & Weath. and *Puccinellia phryganodes* (Trin.) Scribn. & Merr.

###### Specimens examined.

**Canada. Nunavut**: Kitikmeot Region, Victoria Island, Oterkvik Point vicinity, ca. 8 km N of Coronation Gulf coast, 11 km N of point, 68°34'32"N, 112°36'57"W, 25–35 m, 5 July 2008, *Gillespie, Saarela, Consaul & Bull 7570* (ALA, CAN-592376, O); Kitikmeot Region, Victoria Island, vicinity of Nakoyoktok River, 1.5–2 km southwest of outflow of river from large unnamed lake, ca. 18 km ENE of Johansen Bay, 68°38'37"N, 110°42'22"W, 20–30 m, 19 July 2008, *Gillespie, Saarela, Consaul & Bull 8068* (ALA, CAN-593265, MT, O, UBC); Kitikmeot Region, Victoria Island, W end of Johansen Bay at mouth of Mackenzie Creek, 68°36'4"N, 111°21'7"W, 0–20 m, 20 July 2008, *Gillespie, Saarela, Consaul & Bull 8137* (ALTA, BABY, CAN-593267). **Northwest Territories**: Inuvik Region, Victoria Island, NE corner of Boot Inlet, frost boils in *Dryas*-*Arctagrostis* tundra above rocky seashore, 71°28'14.5"N, 117°21'36.7"W, 5 m, 10 July 2010, *Gillespie, Saarela, Doubt, Bull & Sokoloff 9662* (CAN-598332, O); Inuvik Region, Victoria Island, head of Minto Inlet, end of easternmost inlet (N arm), coastal saline flat, 71°31'6.5"N, 115°6'30.4"W, 1–10 m, 25 July 2010, *Gillespie, Saarela, Doubt, Bull & Sokoloff 10243* (CAN-598331).

**Figure 11.**
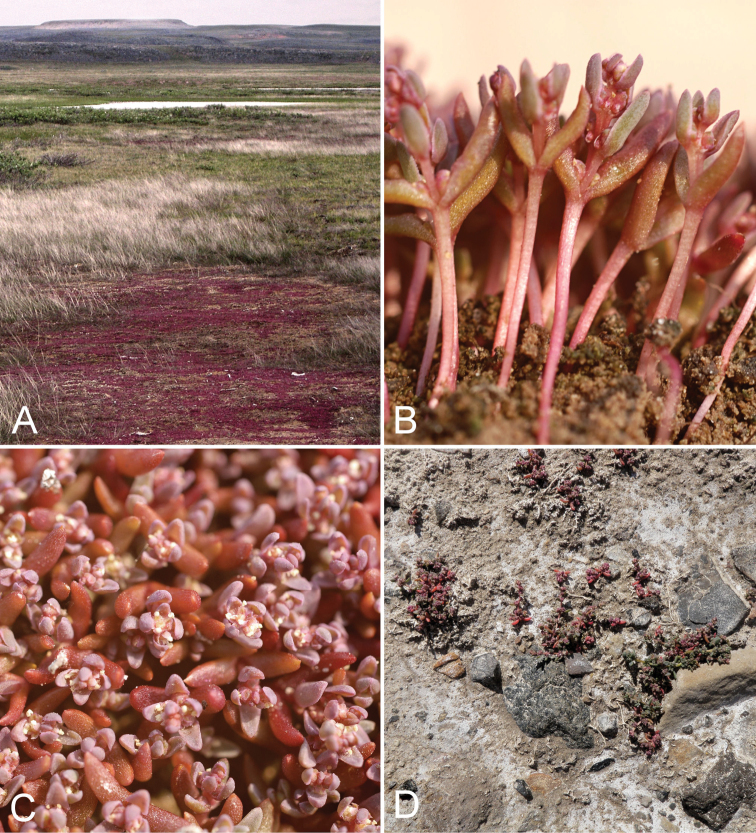
*Suaeda calceoliformis*: **A** habitat **B** habit, profile **C** inflorescences, *Gillespie et al. 8068* **D** habit, *Gillespie et al. 10243*. Photographs by R.D. Bull (**A, B, C**), L.J. Gillespie (**D**).

#### Caryophyllaceae

##### Arenaria humifusa

Taxon classificationPlantaeCaryophyllalesCaryophyllaceae

Wahl.

Fig. 12

###### Common name.

Creeping sandwort

###### Distribution.

Arctic North America–amphi-Atlantic

###### Comments.

Our collections from the Minto Inlet area of Victoria Island represent the first record of the species from the western CAA. Plants were matted, often large, forming loose circular cushions and were found growing on inland sand dunes. Although the species is primarily distributed in the eastern Canadian Arctic and sub-Arctic (south to Nova Scotia) and around Hudson Bay, it is also found scattered on the Northwest Territories and Nunavut mainland south of Victoria Island (specimens at CAN). Porsild and Cody (1980) treated *Arenaria humifusa* in the broad sense including *Arenaria longipedunculata* (see below) and the distribution shown for Alaska, Yukon, and part of the Northwest Territories is that of the latter species. Neither species has previously been recorded for the western Arctic Islands (Porsild and Cody 1980, Aiken et al. 2007). Our Minto Inlet collections have short pedicels (0.5–4 mm long) with mostly very short retrorse hairs (and few scattered glandular hairs), flowers not exserted above the leaves, glabrous sepals and smooth leaf margins, all characteristics of *Arenaria humifusa* s.s.

###### Specimen examined.

**Canada. Northwest Territories**: Inuvik Region, Victoria Island, Sand dunes east of Kuujjua River, 2 km south of lower Kuujjua River, 71°10'4.8"N, 116°27'54"W, 110 m, 16 July 2010, *Gillespie, Saarela, Doubt, Bull & Sokoloff 9882* (ALA, CAN-599149, O); Inuvik Region, Victoria Island, Sand dunes east of Kuujjua River, 2 km south of lower Kuujjua River, 71°10'4.8"N, 116°27'54"W, 110 m, 16 July 2010, *Gillespie, Saarela, Doubt, Bull & Sokoloff 9893* (ALA, CAN-599166, O); Inuvik Region, Victoria Island, sandy bank of Kuujjua River, south of “Fish Lake”, 71°6'43.2"N, 116°6'21.2"W, 74 m, 17 July 2010, *Gillespie, Saarela, Doubt, Bull & Sokoloff 9971* (CAN-599167).

##### Arenaria longipedunculata

Taxon classificationPlantaeCaryophyllalesCaryophyllaceae

Hultén

###### Common name.

Long-stemmed sandwort

###### Distribution.

Arctic-alpine amphi-Beringia–North America

###### Comments.

Our collections are the first records of the species for the CAA and Nunavut. Described by Hultén (1966) from Arctic Alaska, this species was considered conspecific with *Arenaria humifusa* by Porsild and Cody (1980), but has most recently been treated as a separate species (Cody 2000, Hartman et al. 2005, Elven et al. 2011). While considered to have an amphi-Beringian distribution, Elven et al. (2011) suggested that the species may also be present in the CAA and Greenland based on the results of a phylogeographical analysis of AFLP data (Westergaard et al. 2011), a hypothesis supported here. Our collections from southeastern Victoria Island (det. R. Elven) and Baffin Island were identified as this species based on the character combination of long pedicels (10–20 mm) with glandular villous pubescence (not very short retrorse), flowers long-exserted above the leaves, sepals glandular villous basally, and leaf blade margins ciliate proximally (at least sparsely) (Hartman et al. 2005). We found that pedicel length varied among collections and was sometimes shorter than the range given for *Arenaria longipedunculata* (10–20 mm) in Hartman et al. (2005); however, other characters were consistent with our identification. Pedicels are 10–20 mm (*Saarela et al. 2776*) and 5–10 mm long (*Saarela et al. 2477*) on the Baffin Island collections, and 9–12 mm long (*Gillespie et al. 7721*) on the Victoria Island collection (flowers were still in bud with pedicels up to 6 mm long on *Gillespie et al. 8136*). Plants were small and tufted, and were growing in moss on moist to wet riparian meadows on Victoria Island and in mossy tundra at base of slopes or cliffs on Baffin Island.

The ranges of *Arenaria longipedunculata* and *Arenaria humifusa* overlap in the Arctic Islands; indeed we collected both species in the Soper River-Kimmirut area on Baffin Island, and both on Victoria Island but in different localities. In northern Quebec and Newfoundland some large specimens identified as *Arenaria humifusa* appear to approach *Arenaria longipedunculata* in some characters; these robust matted plants have elongate stems with long internodes and pedicels. Further study of this species complex is needed to determine more precisely species boundaries and distributions and to determine if hybrid or introgressed populations exist in the Canadian Arctic.

###### Specimens examined.

**Canada. Nunavut**: Kitikmeot Region, Victoria Island, vicinity of river flowing into Clauston Bay, 3–4 km from river mouth, 69°2'39"N, 113°25'15"W, 10–20 m, 8 July 2008, *Gillespie, Saarela, Consaul & Bull 7721* (CAN-592340); Kitikmeot Region, Victoria Island W end of Johansen Bay at mouth of Mackenzie Creek, 68°36'4"N, 111°21'7"W, 0–20 m, 20 July 2008, *Gillespie, Saarela, Consaul & Bull 8136* (CAN-593142); Qikiqtaaluk Region, Baffin Island, Katannilik Territorial Park Reserve, Soper River, 18.5 km downstream (south) of its confluence with the Livingstone River, 2 km south of Emergency Cabin #8, west side of river, 62°59'20"N, 69°43'41"W, 36 m, 15 July 2012, *Saarela, Gillespie, Sokoloff & Bull 2477* (CAN-601731); Qikiqtaaluk Region, Baffin Island, Kimmirut, west end of Fundo Lake, ca. 2 km west of hamlet, 62°50'44"N, 69°54'6"W, 40 m, 22 July 2012, *Saarela, Gillespie, Sokoloff & Bull 2776* (CAN-601732).

##### Sabulina stricta

Taxon classificationPlantaeCaryophyllalesCaryophyllaceae

(Sw.) Rchb.

Minuartia stricta (Sw.) Hiern

###### Common name.

Bog stitchwort

###### Distribution.

Circumpolar-alpine

###### Comments.

This species was first recorded for the western CAA, on southern Victoria Island, by Thannheiser et al. (2001; no voucher collection located), and is confirmed by our collection. The species is known from Baffin, Southampton, and Coats Islands in the eastern Arctic Islands, and has a scattered distribution across the low Arctic (and north-west alpine areas) from Alaska to Labrador and Greenland. On mainland Nunavut it is currently known only from the Hudson Bay area, and in the Northwest Territories from the vicinity of Great Bear Lake and the Hornaday River (Porsild and Cody 1980, Saarela et al. 2013a). The Victoria Island collections represent a range extension of ca. 400 km northeast of the Northwest Territories populations and ca. 1000 km west of the closest Nunavut population.

This species was previously known as *Minuartia stricta* (Sw.) Hiern (e.g., Porsild and Cody 1980, Rabeler et al. 2005, Aiken et al. 2007) (the name *Sabulina stricta* (Michx.) Small ex Rydb., based on *Arenaria stricta* Michx. [=*Sabulina michauxii* (Fenzl) Dillenb. & Kadereit, a non-Arctic species], is an illegitimate homonym). Recent molecular studies have determined *Minuartia* to be polyphyletic (Harbaugh-Reynaud et al. 2010, Greenberg and Donoghue 2011, Saarela et al. 2013b, Dillenberger and Kadereit 2014). The most comprehensive sampling of the genus was conducted by Dillenberger and Kadereit (2014), who proposed a new classification of the group. The clade to which *Minuartia stricta* belongs (“clade 10”) has been segregated as a distinct genus, *Sabulina* Rchb., with 65 species. *Sabulina* includes four other Canadian Arctic species: *Sabulina dawsonensis* (Britton) Rydb. [syn. *Minuartia dawsonensis* (Britton) House], *Sabulina elegans* (Cham. & Schltdl.) Dillenb. & Kadereit [syn. *Minuartia elegans* (Cham. & Schltdl.) Schischk], *Sabulina rossii* (R.Br.) Dillenb. & Kadereit [syn. *Minuartia rossii* (R.Br.) Graebn.], *Sabulina rubella* (Wahlenb.) Dillenb. & Kadereit [syn. *Minuartia rubella* (Wahlenb.) Hiern.]. Four Canadian Arctic species, *Minuartia biflora* (L.) Schinz & Thell., *Minuartia arctica* (Steven ex Ser.) Graebn., *Minuartia obtusiloba* (Rydb.) House, and *Minuartia yukonensis* Hultén, are part of “clade 6” in Dillenberger and Kadereit (2014), which they recognize as the genus *Cherleria* L., with some 19 species. Combinations for these species in *Cherleria* are not available; we assume they will be published in a revision of *Cherleria* that is noted to be in preparation (Dillenberger and Kadereit 2014, see their Appendix S3). *Cherleria* is distinguished from *Sabulina* by sepals obtuse and oblong (versus acute and linear-lanceolate) (Dillenberger and Kadereit 2014). *Minuartia macrocarpa* (Pursh) Ostenfeld (= *Pseudocherleria macrocarpa* (Pursh) Dillenb. & Kadereit) is part of “clade 3”, which is recognized as the new genus *Pseudocherleria* Dillenb. & Kadereit, with ca. 12 species. *Pseudocherleria* has obtuse sepals, but differs in its long acute multicellular hairs (Dillenberger and Kadereit 2014). *Minuartia groenlandica* (Retzius) Ostenfeld (= *Mononeuria groenlandica* (Retzius) Dillenb. & Kadereit) is part of “clade 5”, recognized as the genus *Mononeuria* Rchb., characterized by an annual or biennial habit and emarginate petals (sometimes absent) twice as long as the sepals (Dillenberger and Kadereit 2014). There are no species of *Minuartia* s.s. in the Canadian Arctic. Of the above species only *Sabulina elegans*, *Sabulina rossii*, *Sabulina rubella*, *Sabulina stricta*, and *Minuartia biflora* occur in the CAA.

*Sabulina stricta* may be distinguished from the closely related and largely sympatric *Sabulina rossii*–*Sabulina elegans* species complex by the presence of branched flowering stems bearing two or more flowers (versus always unbranched and 1-flowered in the latter). Recent molecular evidence suggests that *Sabulina stricta* may be part of this species complex and not easily separable from the genetically diverse species *Sabulina elegans* (Saarela et al. 2013b, S. Leung and L.J. Gillespie, unpubl. data).

###### Specimens examined.

**Canada. Nunavut**: Kitikmeot Region, Victoria Island, rocky hills S of large unnamed lake ca. 18 km ENE of Johansen Bay airstrip, 68°38'43"N, 110°40'9"W, 50–80 m, 14 July 2008, *Gillespie, Saarela, Consaul & Bull 7966* (ALA, CAN-592334, MT, O).

**Figure 12.**
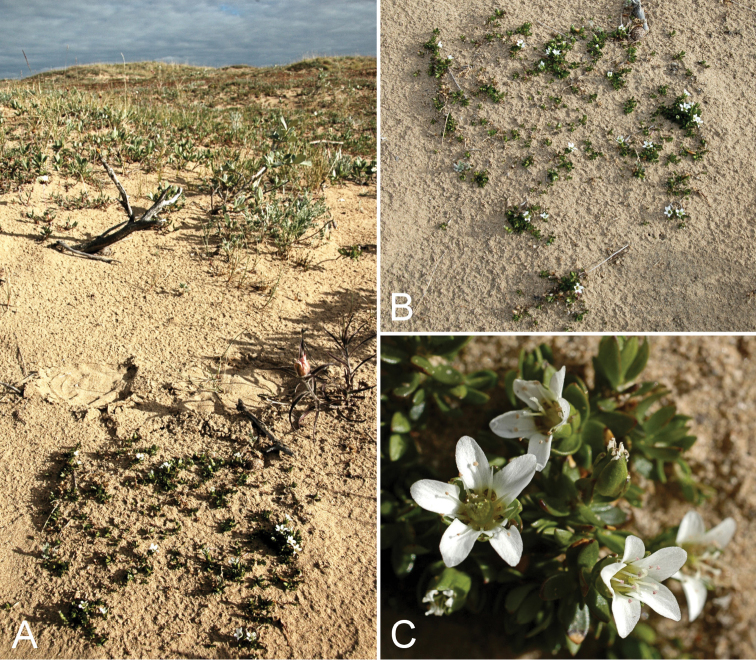
*Arenaria humifusa*: **A** habitat **B** habit **C** flowers, *Gillespie et al. 9882*. Photographs by L.J. Gillespie.

#### Ericaceae

##### Andromeda polifolia

Taxon classificationPlantaeEricalesEricaceae

L.

Fig. 13

###### Common name.

Bog rosemary

###### Distribution.

Circumboreal-polar

###### Comments.

This species was first reported from the Arctic Islands by Thannheiser et al. (2001; no voucher collection located for confirmation), at Johansen Bay on the southern coast of Victoria Island; however, it was not included in Aiken et al. (2007). We collected it at Johansen Bay, confirming its presence there, and along the Soper River on southern Baffin Island, extending the range of this boreal species northwards across the low Arctic islands.

*Andromeda polifolia* has a broad circumboreal-polar distribution, and occurs from Alaska across much of Canada and northern United States to western Greenland (Fabijan 2009). Numerous collections have been reported from the mainland Arctic (Porsild and Cody 1980, Saarela et al. 2013a), including sites south of Coronation Gulf across from our collection site on Victoria Island. On Victoria Island we encountered a single, large population of the species growing in dense moss-sedge mats along the sides of hummocks and polygon ridges in a hummocky, moist to wet sedge meadow on a gentle west-facing slope, in association with *Dryas integrifolia*, *Arctous rubra*, *Vaccinium uliginosum*, Rhododendron tomentosum subsp. decumbens, *Salix reticulata*, *Cassiope tetragona* and *Carex* spp. We collected the species in the Soper River valley, Baffin Island, in a large, wet and hummocky sedge meadow, growing in association with *Carex rariflora*, *Betula glandulosa*, *Salix arctophila*, and *Luzula wahlenbergii*. We observed three patches at this location, one 3 × 2 m, and two smaller ones along the edge of a pond. Our collection in this area was made on the east side of the Soper River; we also observed the species in the area on the west side of the river, but did not collect it there.

Elven et al. (2011) provisionally treat *Andromeda polifolia* as two subspecies—the Eurasian Andromeda polifolia subsp. polifolia and the widespread Andromeda polifolia subsp. pumila V.M. Vinogr. However, due to difficulties in circumscribing diagnostic characters this division is difficult to quantify, and they call for an in depth investigation of this taxon. Fabijan (2009) treats the species as possessing two varieties: the northern boreal-Arctic var. polifolia, and the more southern and eastern var. latifolia Aiton. Our collections would be considered as Andromeda polifolia var. polifolia following this treatment.

###### Specimens examined.

**Canada. Nunavut**: Kitikmeot Region, Victoria Island, slope at S end of unnamed lake, ca. 20 km ENE of Johansen Bay airstrip, 68°36'27"N, 110°40'35"W, 30–50 m, 16 July 2008, *Gillespie, Saarela, Consaul & Bull 8002* (ALA, BABY, CAN-592360, MT, O, UBC); Qikiqtaaluk Region, Baffin Island, Katannilik Territorial Park Reserve, Soper River valley, E bank, large sedge meadow with several small ponds ca. 12.5 km south of Mount Joy, 0.5 km south of Group/Warden Cabin #7, 63°9'35"N, 69°40'3"W, 41 m, 7 July 2012, *Saarela, Gillespie, Sokoloff & Bull 2186* (ALA, CAN-601935, MO, MT, O, US, WIN).

##### Orthilia secunda subsp. obtusata

Taxon classificationPlantaeEricalesEricaceae

(Turcz.) Böcher

Pyrola secunda var. obtusata Turcz.

###### Common names.

One-sided wintergreen, nodding wintergreen

###### Distribution.

Disjunct circumpolar (excluding Europe)

###### Comments.

This is the first collection of the species from the eastern CAA; previously it had been collected in the western Arctic Islands at two localities on Victoria Island (Aiken et al. 2007), where we also collected it from a third locality, and one on Banks Island (Porsild and Cody 1980; not mapped in Aiken et al. 2007). In the eastern North American Arctic, this species is known from western Greenland (Böcher et al. 1968) and Ungava Bay, Quebec (Porsild and Cody 1980), thus our collection fills in a distributional gap in the general area between these sites, extending the range to southern Baffin Island. Our collection on Baffin Island comes from a single population encountered along the Soper River. This small population was found growing abundantly in a wet snowbed community with *Cassiope tetragona*, *Vaccinium uliginosum*, and *Salix reticulata*. On Victoria Island, we found this species growing in a similar habitat: a wet sedge meadow formed by a drainage between two lakes, associated with Carex aquatilis var. minor, *Eriophorum angustifolium*, *Dryas integrifolia*, *Salix reticulata*, *Salix glauca*, and *Pedicularis albolabiata* (Hultén) Kozhevn.

Elven et al. (2011) recognize this taxon at the species level as *Orthilia obtusata* (Turcz.) H. Hara, a circumpolar plant distinct from the mostly circumboreal *Orthilia secunda* (L.) House. Freeman (2009) treats North American material as widely variable, and synonymises subsp. obtusata under *Orthilia secunda*. As there are distinctions between the taxa, most pronounced in Eurasia, we follow Aiken et al. (2007), and treat the Arctic taxon as Orthilia secunda subsp. obtusata, an approach intermediate to those of Elven et al. (2011) and Freeman (2009).

###### Specimen examined.

**Canada. Nunavut**: Kitikmeot Region, Victoria Island, Johansen Bay, 18 km ENE of airstrip, Nakoyoktok River at outflow of large unnamed lake, 62°39'25"N, 110°42'30"W, 20–30 m, 18 July 2008, *Gillespie, Saarela, Consaul & Bull 8036* (ALA, CAN-592359); Qikiqtaaluk Region, Baffin Island, Katannilik Territorial Park Reserve, Soper River, 18.5 km downstream (S) of its confluence with the Livingstone River, 2 km S of Emergency Cabin #8, W side of river, 62°59'28"N, 69°43'30"W, 67 m, 15 July 2012, *Saarela, Gillespie, Sokoloff & Bull 2489* (CAN-601915).

**Figure 13.**
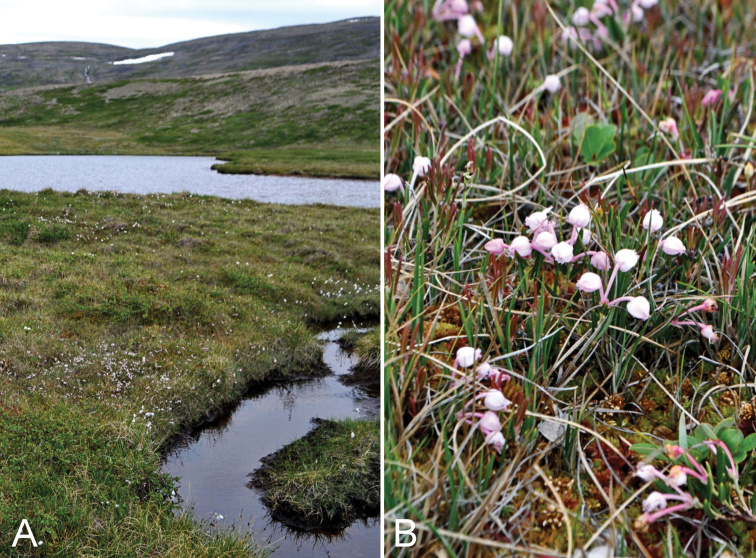
*Andromeda polifolia*: **A** habitat **B** habit, *Saarela et al. 2186*. Photographs by P.C. Sokoloff.

#### Fabaceae

##### Oxytropis deflexa subsp. foliolosa

Taxon classificationPlantaeFabalesFabaceae

(Hook.) Cody

Fig. 14

###### Common name.

Pendant-pod oxytrope, pendant-pod locoweed

###### Distribution.

Arctic-alpine North America

###### Comments.

This is the first record of this taxon from the western CAA—the only populations known on the islands previously occur on southeastern Baffin Island near Kimmirut, Iqaluit, and on the Hall Peninsula (Aiken et al. 2007), and we made four additional collections in Katannilik Territorial Park on southern Baffin Island. On the mainland Arctic, this taxon has been collected in the vicinity of Coronation Gulf south of Victoria Island (67°45'N, 111°57'W) (Macoun and Holm 1921). Subspecies *foliolosa* is common in the boreal forest of Yukon and Alaska, extends south along the Rocky Mountains to Colorado, and occurs along the coast in northern Ontario and Quebec (Welsh 1974, Porsild and Cody 1980, Blondeau and Cayouette 2002). A collection (*Baldwin 1997*, CAN-203476) from the vicinity of Longstaff Bluff (68°58'N, 47°57'W) on the west coast of Baffin Island is included in the range map for this species in Porsild (1957). However, Porsild re-identified this collection to *Astragalus alpinus* L. in 1959 (a determination with which we agree) and, while the dot on the map is erroneously reproduced in Porsild and Cody (1980), it is correctly omitted from the map in Aiken et al. (2007). We encountered only one small population on Victoria Island, consisting of six individuals growing on a rocky river flat at the edge of a low thicket of *Salix alaxensis* (Andersson) Coville, associated with *Chamerion latifolium*, *Astragalus alpinus*, *Castilleja elegans* Malte and *Saxifraga tricuspidata* Rottb. This collection extends the range of this species north by approximately 300 kilometers from Coronation Gulf, where J. Cox collected it during the Canadian Arctic Expedition 1913–1918 (Macoun and Holm 1921, Polunin 1940).

Isely (1998) synonymized this taxon (as var. foliolosa (Hook.) Barneby) under var. deflexa, but did so only taking into account material from continental United States, excluding Alaska. Here we follow Cody (1994) and Aiken et al. (2007) and recognize subsp. foliolosa as a discrete taxon in North America. In a pan-Arctic context, Elven et al. (2011) suggested that this taxon may be synonymous with the Russian Oxytropis deflexa subsp. dezhnevii (Jurtz.) Jurtz. Further work is needed to clarify the statuses of these taxa, but the Russian name would have priority if these taxa were synonymized.

###### Specimens examined.

**Canada. Northwest Territories**: Inuvik Region, Victoria Island, River valley at N head of Minto Inlet, ca. 3 km from inlet, 71°33'46.7"N, 115°22'45.1"W, 24 m, 23 July 2010, *Gillespie, Saarela, Doubt, Bull & Sokoloff 10129* (CAN-598345). **Nunavut**: Qikiqtaaluk Region, Baffin Island, Katannilik Territorial Park Reserve, Soper River, west side, ca. 44.5 km south of Mount Joy along river, ca. 17 km south of confluence with Livingstone River, 62°57'51"N, 69°47'53"W, 33 m, *Saarela, Gillespie, Sokoloff & Bull 2504* (ALA, ALTA, CAN-601898, MO, NFM, UTC, UTU, US, UVIC, WIN); Qikiqtaaluk Region, Baffin Island, Katannilik Territorial Park Reserve, Soper Falls, south side of Soper Lake, just southeast of Soper Falls, 62°54'8"N, 69°50'42"W, 6 m, 17 July 2012, *Saarela, Gillespie, Sokoloff & Bull 2530* (ALA, CAN-601901); Qikiqtaaluk Region, Baffin Island, Kimmirut, north end of Fundo Lake below Taqaiqsirvik Territorial Park, 62°50'50"N, 69°53'40"W, 35 m, 20 July 2012, *Saarela, Gillespie, Sokoloff & Bull 2658* (CAN-601900); Qikiqtaaluk Region, Baffin Island, Pleasant Inlet, ca. 10 km south of Reversing Falls at end of Soper Lake, west of Kimmirut, west side of inlet 62°47'22"N, 69°59'51"W, 10–25 m, 21 July 2012, *Saarela, Gillespie, Sokoloff & Bull 2714* (ALA, CAN-601899, MT, O, UBC).

**Figure 14.**
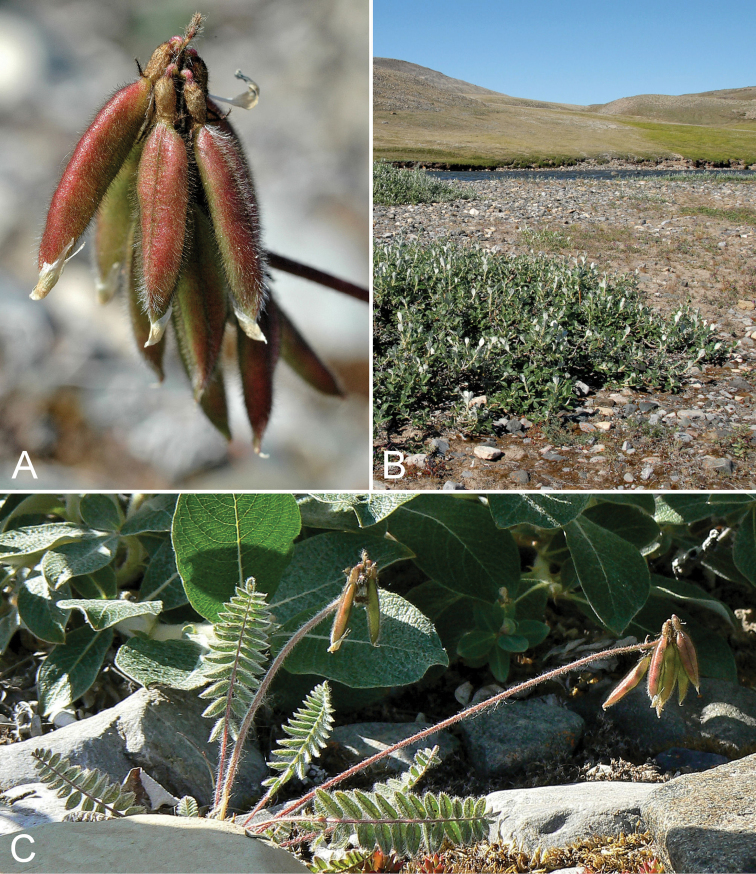
Oxytropis deflexa subsp. foliolosa: **A** fruits **B** habitat **C** habit, *Gillespie et al. 10129*. Photographs by R.D. Bull (**A**), L.J. Gillespie (**B**), P.C. Sokoloff (**C**).

#### Lentibulariaceae

##### Pinguicula vulgaris

Taxon classificationPlantaeLamialesLentibulariaceae

L.

Fig. 15

###### Common name.

Butterwort

###### Distribution.

Nearly circumboreal-polar

###### Comments.

This species was first reported from the western Arctic Islands by Thannheiser et al. (2001; no voucher collection located for confirmation) at Johansen Bay on Victoria Island (they incorrectly considered their record as the first for the whole CAA). We collected this species at Johansen Bay and Clauston Bay on southwestern Victoria Island and from three sites in the Minto Inlet area on northwestern Victoria Island, extending the northern range of this low Arctic species. On Victoria Island the species was sometimes locally common and populations were scattered mostly in moist to wet meadows on river flats. Two nearby populations were discovered in rocky tundra on the top of a plateau south of Minto Inlet; the larger population (*Gillespie et al. 9967*) comprised about 50 plants scattered in a moist depression in a boulder field adjacent to an *Eriophorum* meadow above the head of a canyon. The species was previously known in the CAA based on four collections from southeastern Baffin Island (Porsild 1957, Porsild and Cody 1980, Aiken et al. 2007). We collected it at six sites there, in the vicinity of Kimmirut (where previously known) and from five sites along the Soper River (one previous collection known).

###### Specimens examined.

**Canada. Nunavut**: Kitikmeot Region, Victoria Island, vicinity of river flowing into Clauston Bay, 3–4 km from river mouth, 69°2'39"N, 113°25'15"W, 10–20 m, 8 July 2008, *Gillespie, Saarela, Consaul, & Bull 7718* (ALA, CAN-592385, MT, O); Kitikmeot Region, Victoria Island, W end of Johansen Bay at mouth of Mackenzie Creek, 68°36'4"N, 111°21'7"W, 0–20 m, 20 July 2008, *Gillespie, Saarela, Consaul & Bull 8132* (CAN-592384); Qikiqtaaluk Region, Baffin Island, Katannilik Territorial Park Reserve, Soper River, west bank, near confluence with Livingstone River, crystalline limestone ridge just north of confluence, 63°6'38"N, 69°44'14"W, 100m, 10 July 2012, *Saarela, Gillespie, Sokoloff & Bull 2264* (CAN-601974); Qikiqtaaluk Region, Baffin Island, Katannilik Territorial Park Reserve, Livingstone River (major tributary of Soper River), north side, near confluence with Soper River, ca. 0.5 km northwest of Livingstone Falls, 63°6'32"N, 69°44'38"W, 141m, 12 July 2012, *Saarela, Gillespie, Sokoloff & Bull 2381* (CAN-601972, MO); Qikiqtaaluk Region, Baffin Island, Katannilik Territorial Park Reserve, Soper River, 18.5 km downstream (south) of its confluence with the Livingstone River, 2 km south of Emergency Cabin #8, west side of river, 62°59'17"N, 69°43'47"W, 60m, 15 July 2012, *Saarela, Gillespie, Sokoloff & Bull 2478* (ALA, CAN-601970, WIN); Qikiqtaaluk Region, Baffin Island, Katannilik Territorial Park Reserve, Soper Falls, south side of Soper Lake, just southeast of Soper Falls, 62°54'1"N, 69°50'48"W, 6 m, 17 July 2012, *Saarela, Gillespie, Sokoloff & Bull 2531* (CAN-601973); Qikiqtaaluk Region, Baffin Island, Katannilik Territorial Park Reserve, Soper Falls, north side of Soper River, 62°54'35"N, 69°50'43"W, 20m, 18 July 2012, *Saarela, Gillespie, Sokoloff & Bull 2565* (CAN-601975); Qikiqtaaluk Region, Baffin Island, Kimmirut, northwest end of Fundo Lake, ca. 2 km west of hamlet, 62°50'36"N, 64°54'10"W, 30m, 22 July 2012, *Saarela, Gillespie, Sokoloff & Bull 2787* (CAN-601971). **Northwest Territories**: Inuvik Region, Victoria Island, plateau above head of enclosed valley S of “Fish Lake” on lower Kuujjua River, 71°10'44.3"N, 116°27'11.9"W, 120 m, 16 July 2010, *Gillespie, Saarela, Doubt, Bull & Sokoloff 9878* (CAN-599229); Inuvik Region, Victoria Island, wet rocky tundra on plateau above head of enclosed valley S of “Fish Lake” on lower Kuujjua River, 71°10'14.2"N, 116°27'29.1"W, 100 m, 16 July 2010, *Gillespie, Saarela, Doubt, Bull & Sokoloff 9880* (CAN-599258); Inuvik Region, Victoria Island, sandy bank of Kuujjua River S of “Fish Lake”, 71°6'43.2"N, 116°6'21.2"W, 70 m, 17 July 2010, *Gillespie, Saarela, Doubt, Bull & Sokoloff 9967* (CAN-599230, O).

**Figure 15.**
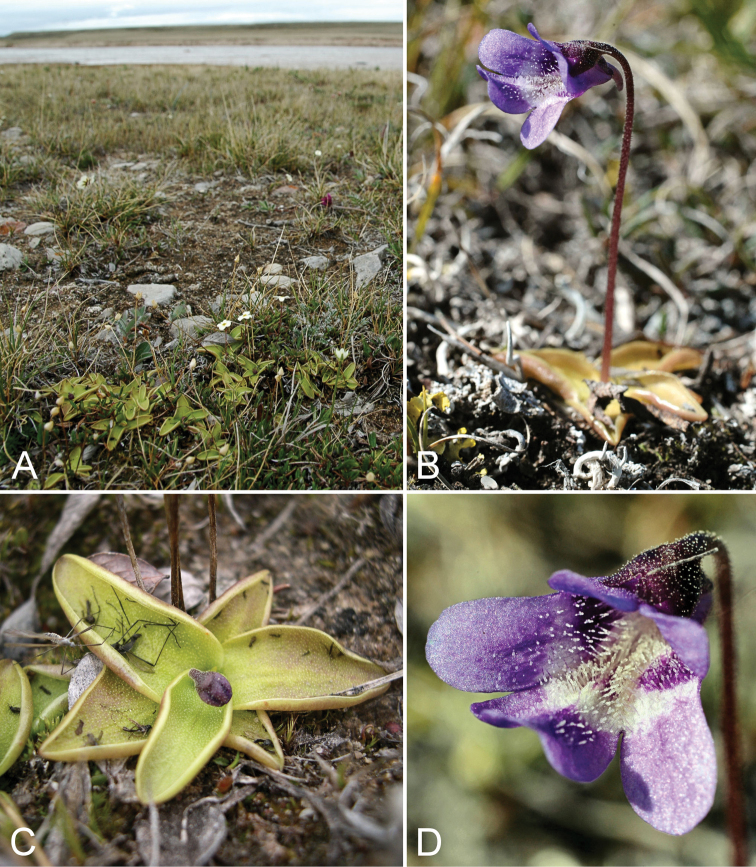
*Pinguicula vulgaris*: **A** habitat, *Gillespie et al. 7718* **B** habit, *Gillespie et al. 8983b* **C** leaves, *Gillespie et al. 7718* **D** flower, *Gillespie et al. 8983b*. Photographs by R.D. Bull.

##### Utricularia ochroleuca

Taxon classificationPlantaeLamialesLentibulariaceae

R.W. Hartm.

Fig. 16

###### Common name.

Yellowish-white bladderwort

###### Distribution.

Circumboreal

###### Comments.

This is the first collection of this genus and species for the CAA, and the first record of the species for Nunavut. Although broadly distributed across boreal Canada (Porsild and Cody 1980) and reported from nine states (NatureServe 2014), the species is rare in North America, with only 25 localities known prior to this collection (G. Crow, pers. comm. 2014). This uncommon plant has previously been collected at two Arctic localities in Canada: Richards Island, Mackenzie Delta, Northwest Territories (*Porsild 7076*, CAN-99617) and along the eastern coast of Hudson Bay in northern Quebec (Porsild and Cody 1980). The species is also present in west Greenland (Elven et al. 2011). Despite previous reports (Taylor 1989), this taxon has recently been excluded from the flora of Alaska (Alaska Natural Heritage Program 2014).

We encountered a single population of *Utricularia ochroleuca* on southern Baffin Island, forming a dense floating mat along the bottom of a shallow muddy pond in a wet sedge meadow comprised of *Carex bigelowii* Torr. ex Schwein., *Carex chordorrhiza* Ehrh. ex L. f., *Carex holostoma*, *Betula glandulosa*, Arctagrostis latifolia subsp. latifolia, *Eriophorum vaginatum* L. and Eriophorum scheuchzeri subsp. scheuchzeri. This population was uniformly sterile—no conspicuous emergent flowers were seen. This pattern is seen in many species of *Utricularia* above the treeline (Porsild and Cody 1980), particularly *Utricularia ochroleuca* (G. Crow, pers. comm. 2014). This species may be more common in the low Arctic than herbarium records suggest and should be looked for carefully.

###### Specimens examined.

**Canada. Nunavut**: Qikiqtaaluk Region, Baffin Island, Katannilik Territorial Park Reserve, Soper River, 18.5 km downstream (S) of its confluence with the Livingstone River, 1.5 km S of Emergency Cabin #8, E bank of river, 62°58'45"N, 69°43'1"W, 23 m, 15 July 2012, *Saarela, Gillespie, Sokoloff & Bull 2464* (ALA, ALTA, CAN-601976, MT, O, NY, UBC, US, WIN).

**Figure 16.**
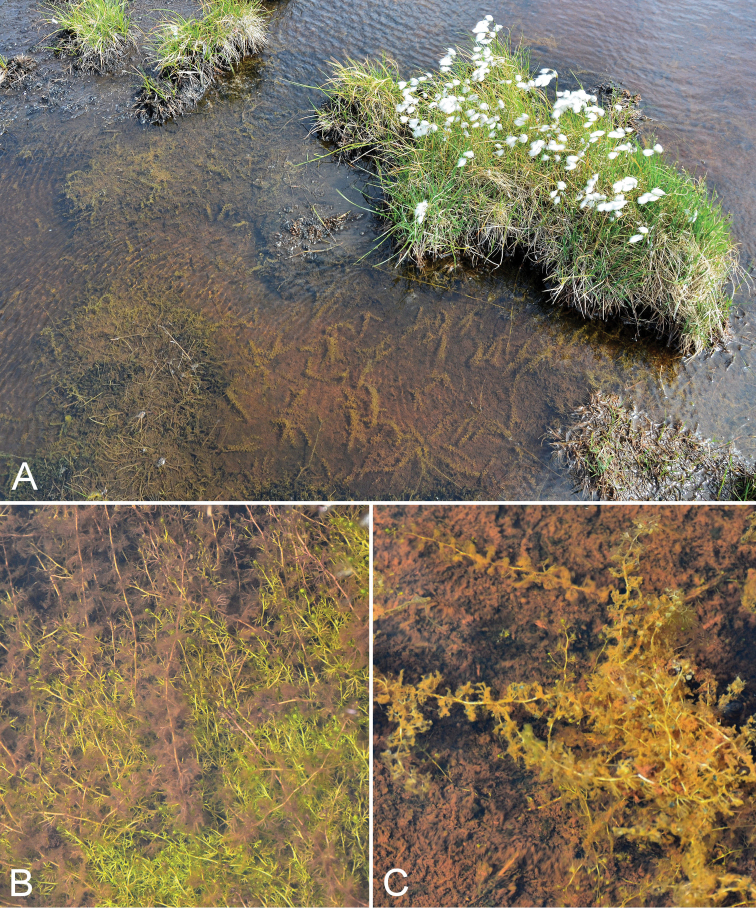
*Utricularia ochroleuca*: **A** habitat **B** habit **C** habit showing branches with bladders, *Saarela et al. 2464*. Photographs by R.D. Bull.

#### Primulaceae

##### Primula egaliksensis

Taxon classificationPlantaeEricalesPrimulaceae

Wormsk.

Fig. 17

###### Common name.

Greenland primrose

###### Distribution.

Arctic-alpine amphi-Beringia–North America

###### Comments.

Our collections are the first for this species in the CAA. This species commonly occurs along lakeshores and riverbeds in tundra and alpine regions of Canada, Greenland and the United States (Porsild and Cody 1980, Kelso 2009), and is known from both Ungava Bay to the south and Greenland to the west of southeastern Baffin Island where our collections were made. One population collected was found in a moist mossy depression among rocks in a disturbed site near the Kimmirut boat landing on Soper Lake, associated with *Chamerion latifolium*, *Bistorta vivipara* and Cardamine pratensis subsp. angustifolia (Hook.) O.E. Schultz. The second population was on moist mossy ground among rocky outcrops on a small island, with *Leymus mollis*, *Juncus arcticus*, *Dupontia fisheri*, *Puccinellia phryganodes*, Potentilla anserina subsp. egedei (Wormsk. ex Hornem.) Hiitonen and *Saxifraga caespitosa* L. Similar in size and appearance to *Potentilla stricta* Hornem., a largely sympatric species that is found in the CAA on Banks Island and Victoria Island, *Primula egaliksensis* is distinguished by its non-farinose flowering stem (versus farinose at least at the apex), abruptly petiolate leaves, and calyx base that is less prominently saccate and never auriculate (Kelso 2009, Saarela et al. 2013a).

###### Specimens examined.

**Canada. Nunavut**: Qikiqtaaluk Region, Baffin Island, Soper Lake, SE corner, Kimmirut boat landing, 62°51'45"N, 69°52'56"W, 16 m, 19 July 2012, *Saarela, Gillespie, Sokoloff & Bull 2606* (CAN-601987, COCO); Qikiqtaaluk Region, Baffin Island, Katannilik Territorial Park Reserve, small unnamed island on Soper Lake (Eider duck colony), 62°53'6"N, 69°53'18"W, 9 m, 19 July 2012, *Saarela, Gillespie, Sokoloff & Bull 2640* (CAN-601986).

**Figure 17.**
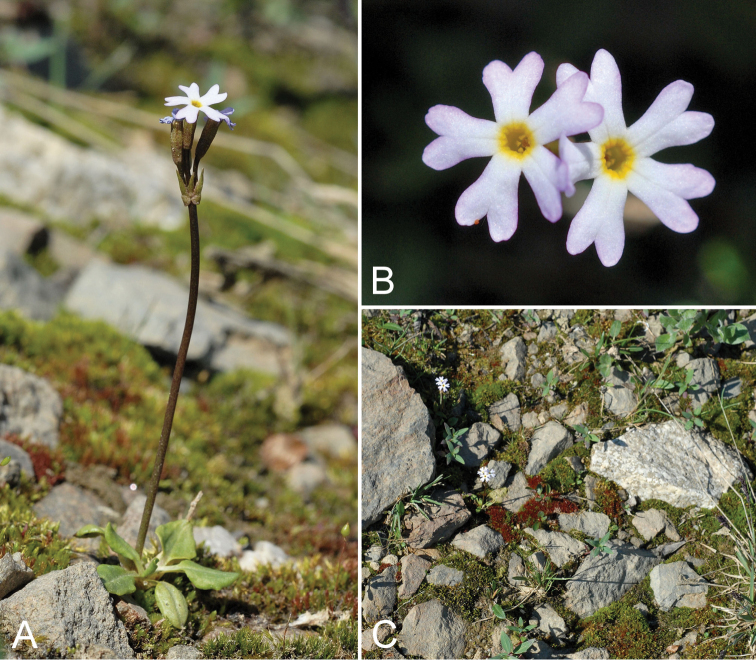
*Primula egaliksensis*: **A** habit **B** inflorescence **C** habitat, *Saarela et al. 2606*. Photographs by L.J. Gillespie.

#### Ranunculaceae

##### Coptidium × spitsbergense

Taxon classificationPlantaeRanunculalesRanunculaceae

(Hadač) Luferov & Prob.

Fig. 18

Ranunculus × spitsbergensis Hadač

###### Common name.

Spitzbergen buttercup

###### Distribution.

Disjunct circumpolar

###### Comments.

Our collections are the first of this species for the CAA. The species is considered to be a sterile triploid hybrid between *Coptidium lapponicum* (L.) Rydb. and *Coptidium pallasii* (Schltdl.) Tzvelev, and exhibits an intermediate morphology and habitat preference (Cody et al. 1988, Elven and Murray 2008). All three species were previously treated within *Ranunculus* L. (Porsild 1957, Porsild and Cody 1980, Cody et al. 1988, Whittemore 1997, Aiken et al. 2007), but they differ both genetically and morphologically (presence of thick white underground stems, fragrant flowers, three sepals, spongy tissue in achene) from other members of the genus (Hörandl et al. 2005). Coptidium × spitsbergense, also known from Svalbard and the Russian Arctic, was first recorded in North America by Cody et al. (1988) from one site in southern mainland Nunavut, and four sites in northwestern Arctic Quebec. The hybrid is most similar in habit and leaf morphology to *Coptidium pallasii*, but differs in its smaller, pale yellow flowers. The taxon was not treated by Whittemore (1997) for North America.

Coptidium × spitsbergense was found at two sites in the Soper River valley growing in sedge meadows, in wet moss adjacent to ponds. Associates at the first site (*Saarela et al. 2194*) include *Carex bigelowii* and *Salix arctophila*, at the second site *Betula glandulosa*, *Empetrum nigrum*, *Eriophorum angustifolium*, *Eriophorum scheuchzeri*, Rhododendron tomentosum subsp. decumbens, *Carex* spp. and *Salix* sp. Only one parent, *Coptidium lapponicum*, was found nearby at the *Saarela et al. 2419* site (parents were not looked for at the other site), growing scattered in moist mossy tundra. The other parent, *Coptidium pallasii*, has not been collected in the Soper River valley and was not observed during our fieldwork there, but one older collection is known from the vicinity of Kimmirut (*Polunin 1173*, CAN; Aiken et al. 2007). Elsewhere the hybrid species is also often found in the absence of one (usually *Coptidium pallasii*) or even both parents. In Svalbard it is more common than either parent and occurs in large stands usually in the absence of one or both parents (Elven and Murray 2008, http://svalbardflora.no/). Cody et al. (1988) recorded *Coptidium lapponicum* as present at all five sites in Canada, and *Coptidium pallasii* as present at only two sites, both in northern Quebec.

Throughout its range fruiting specimens have not been observed. Plants are assumed to be spread mainly by bird dispersal of stem-shoot fragments (Elven and Murray 2008, Elven et al. 2011). However, Cody et al. (1988) considered there to be no evidence for long distance dispersal and suggested that separate hybridization events occurred at each locality sometime in the past.

###### Specimens examined.

**Canada. Nunavut**: Qikiqtaaluk Region, Baffin Island, Katannilik Territorial Park Reserve, Soper River valley, W bank, ca. 12 km S of Mount Joy, meadow along river opposite Group/Warden Cabin #7, 63°9'50"N, 69°39'55"W, 40 m, 8 July 2012, *Saarela, Gillespie, Sokoloff & Bull 2194* (ALA, CAN-602059, O); Qikiqtaaluk Region, Baffin Island, Katannilik Territorial Park Reserve, Soper River, 18.5 km downstream (S) of its confluence with the Livingstone River, 2 km S of Emergency Cabin #8, E bank of river, 62°59'2"N, 69°43'1"W, 20 m, 14 July 2012, *Saarela, Gillespie, Sokoloff & Bull 2419* (ALA, CAN-602060, MT, O, WIN).

**Figure 18.**
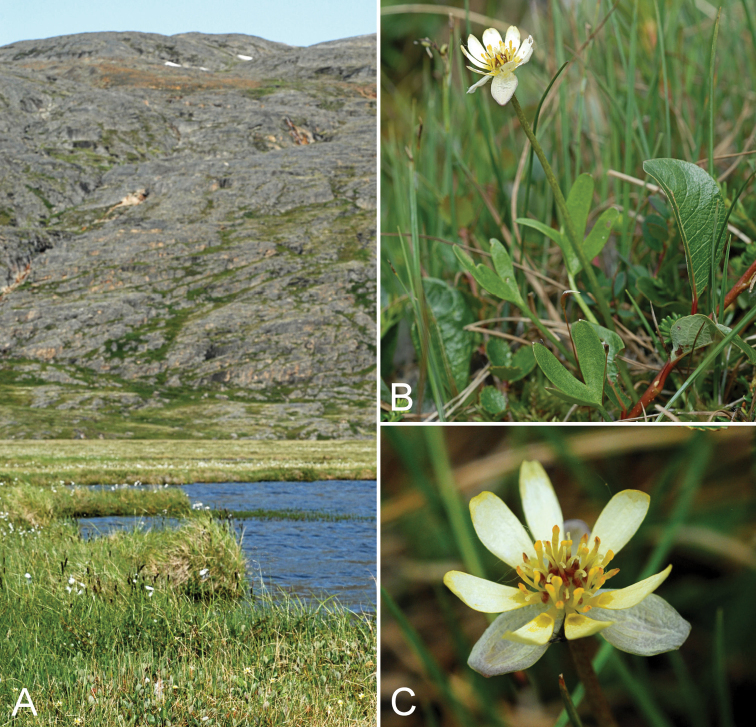
Coptidium × spitsbergense: **A** habitat **B** habit **C** flower, *Saarela et al. 2419*. Photographs by L.J. Gillespie.

#### Salicaceae

##### Salix arctophila

Taxon classificationPlantaeMalpighialesSalicaceae

Cockerell ex A. Heller

###### Common name.

Northern willow

###### Distribution.

Arctic North America

###### Comments.

This is the first record of this species for the western CAA. This sub-Arctic–low Arctic species is distributed from northeastern Alaska to Greenland and south to Maine in the alpine zone (Argus 2007). Its range on the Northwest Territories mainland extends to the coast immediately adjacent to where we collected it at Oterkvik Point (Porsild and Cody 1980, Argus 2007). Previous collections in the Arctic Islands have only been made on Baffin Island and Southampton Island in the eastern CAA (Aiken et al. 2007).

###### Specimen examined.

**Canada. Nunavut**: Kitikmeot Region, Victoria Island, Oterkvik Point vicinity, 9–10 km N of Coronation Gulf coast, 12–13 km N of point, 68°36'23"N, 112°34'7"W, 50–60 m, 4 July 2008, *Gillespie, Saarela, Consaul & Bull 7511* (CAN-592250, O, ALA, MT).

##### Salix fuscescens

Taxon classificationPlantaeMalpighialesSalicaceae

Andersson

###### Common name.

Alaska bog willow

###### Distribution.

Arctic Asia (NE)–amphi-Beringia–North America

###### Comments.

Our collections are the first records for the eastern CAA. Aiken et al. (2007) reported the species in the CAA from a single collection on Victoria Island (*Lambert s. n.*, CAN-52349). Argus (2007) mapped the species as occurring in the low Arctic from Alaska to western Hudson Bay, including southern Victoria Island, King William Island and the lower Boothia Peninsula. Our collections represent an eastern range extension of some 900 km for the species, with respect to the map in Argus (2007).

###### Specimens examined.

**Canada. Nunavut**: Qikiqtaaluk Region, Baffin Island, Katannilik Territorial Park Reserve, Livingstone River (major tributary of Soper River), north side, near confluence with Soper River, 63°06'30"N, 69°44'02"W, 50 m, 12 July 2012, *Saarela, Gillespie, Sokoloff, Bull 2361* (CAN-601675), *2362* (CAN-601674).

#### Saxifragaceae

##### Saxifraga eschscholtzii

Taxon classificationPlantaeSaxifragalesSaxifragaceae

Sternb.

###### Common name.

Eschscholtz’s saxifrage

###### Distribution.

Amphi-Beringia

**Comments**: Collected on Bathurst Island by S. Edlund in 1975, this is only the second collection of this species from the CAA, and the first record from Nunavut. Edlund’s collection was shelved in the backlog of the National Herbarium of Canada for nearly 40 years, and was only recently uncovered. However, its significance as a new record was noted on the newsprint accompanying the specimen, indicating its importance was apparent to the collector.

Though long known from the alpine tundra of northern Yukon and Alaska (Cody 2000), the 1968 collection on Prince Patrick Island (mapped in Porsild and Cody 1980 and Aiken et al. 2007)—the first record for the Arctic Islands—extended the range of this species northeastwards by over 1000 km. The second collection on Bathurst Island pushes this species a further 500 km east in the CAA. The apparent gaps in this species distribution may be explained by its habit: when not in flower, it can resemble either the very common *Saxifraga oppositifolia* L. or a lichen (Aiken et al. 2007); either scenario could account for the paucity of collections from the CAA.

###### Specimens examined.

**Canada. Northwest Territories**: Inuvik Region, Prince Patrick Island, Green Bay, gravelly slopes with northern exposure, 76°33'46"N, 118°51'28"W, 7 July 1968, *Kuc s. n.* (CAN-385465). **Nunavut**: Qikiqtaaluk Region, Bathurst Island, Bracebridge Inlet, GSC [Geological Survey of Canada] Site, 75°35'N, 101°00'W, 1 July 1975, *Edlund 41* (CAN-605793).

##### Saxifraga rivularis subsp. arctolitoralis

Taxon classificationPlantaeSaxifragalesSaxifragaceae

(Jurtz. & V.V. Petrovsky) M.H. Jørg. & Elven

###### Common name.

Alpine brook saxifrage

###### Distribution.

Arctic amphi-Beringia–North America

###### Comments.

This collection (det. R. Elven and L.J. Gillespie) represents the first record of *Saxifraga rivularis* L. as currently circumscribed from the western CAA and the first record of Saxifraga rivularis subsp. arctolitoralis from the CAA. Porsild (1957) and Porsild and Cody (1980) previously treated the species in a broader sense and included plants now treated under *Saxifraga hyperborea* R.Br., a circum-Arctic species (all collections of *Saxifraga rivularis* s.l. mapped by them from the western Arctic Islands are now considered *Saxifraga hyperborea*). As treated by Aiken et al. (2007), *Saxifraga rivularis* is restricted to the eastern CAA, while *Saxifraga hyperborea* is widespread across the CAA; the two species are easily distinguished by the presence of stolons only in *Saxifraga rivularis*. Our collection fills in a distribution gap in the widely disjunct amphi-Atlantic–amphi-Beringian distribution of *Saxifraga rivularis*.

Two subspecies have recently been recognized in Saxifraga rivularis: subsp. rivularis with an amphi-Atlantic distribution (and widespread in the eastern CAA) and subsp. arctolitoralis with an amphi-Beringian distribution (Jørgensen et al. 2006, Brouillet and Elvander 2009, Elven et al. 2011). Jørgensen et al. (2006), Aiken et al. (2007) and Brouillet and Elvander (2009) considered subsp. arctolitoralis as present in Alaska, but not known from Canada. More recently, Westergaard et al. (2010) presented molecular evidence for the presence of subsp. arctolitoralis on southeastern Baffin Island and Greenland, suggesting long distance dispersal from Beringia in the post-glacial period. Elven et al. (2011) consider subsp. arctolitoralis as present also in the Yukon and the Mackenzie Delta area of the Northwest Territories and mention that there are also plants from Hudson Bay and northern Quebec and Labrador conforming in both DNA and morphology to the subspecies. The two subspecies may be distinguished by the following key (adapted from Jørgensen et al. 2006 and Brouillet and Elvander 2009):

**Table:** 

1	Hypanthium densely covered with long stipitate glandular hairs, 0.3–0.6 mm long; flowering stem glabrous or sparsely hairy	**Saxifraga rivularis subsp. arctolitoralis**
–	Hypanthium sparsely covered with short stipitate glandular hairs, 0.1–0.3 mm long; flowering stem sparsely to densely hairy	**Saxifraga rivularis subsp. rivularis**

###### Specimens examined.

**Canada. Nunavut**: Kitikmeot Region, Victoria Island, Murray Point, W side of Wilbank Bay, 68°35'34"N, 110°18'24"W, 20–30 m, 21 July 2008, *Gillespie, Saarela, Consaul & Bull 8174* (ALA, ALTA, BABY, CAN-592397, MT, O, UBC).

## Supplementary Material

XML Treatment for Cryptogramma stelleri

XML Treatment for Carex bicolor

XML Treatment for Carex brunnescens (Pers.) Poir. subsp. brunnescens

XML Treatment for Eriophorum brachyantherum

XML Treatment for Luzula wahlenbergii

XML Treatment for Triglochin palustris

XML Treatment for Corallorhiza trifida

XML Treatment for Platanthera obtusata (Banks ex Pursh) Lindl. subsp. obtusata

XML Treatment for Calamagrostis stricta subsp. groenlandica

XML Treatment for Hordeum jubatum L. subsp. jubatum

XML Treatment for Leymus innovatus subsp. velutinus

XML Treatment for Leymus mollis (Trin.) Pilg. subsp. mollis

XML Treatment for Puccinellia banksiensis

XML Treatment for Stuckenia vaginata

XML Treatment for Suaeda calceoliformis

XML Treatment for Arenaria humifusa

XML Treatment for Arenaria longipedunculata

XML Treatment for Sabulina stricta

XML Treatment for Andromeda polifolia

XML Treatment for Orthilia secunda subsp. obtusata

XML Treatment for Oxytropis deflexa subsp. foliolosa

XML Treatment for Pinguicula vulgaris

XML Treatment for Utricularia ochroleuca

XML Treatment for Primula egaliksensis

XML Treatment for Coptidium × spitsbergense

XML Treatment for Salix arctophila

XML Treatment for Salix fuscescens

XML Treatment for Saxifraga eschscholtzii

XML Treatment for Saxifraga rivularis subsp. arctolitoralis

## Appendix

**Table:** Appendix

CAN accession number	Taxon, collector and collector no.	URL
CAN-601315	*Cryptogramma stelleri*. *Saarela, Gillespie, Sokoloff & Bull 2774.*	http://dx.doi.org/10.6084/m9.figshare.1408637
CAN-592505	*Carex bicolor*. *Gillespie, Saarela, Consaul & Bull 8118.*	http://dx.doi.org/10.6084/m9.figshare.1408638
CAN-601449	Carex brunnescens subsp. brunnescens. *Saarela, Gillespie, Sokoloff & Bull 2232.*	http://dx.doi.org/10.6084/m9.figshare.1408636
CAN-601450	Carex brunnescens subsp. brunnescens. *Saarela, Gillespie, Sokoloff & Bull 2346.*	http://dx.doi.org/10.6084/m9.figshare.1408639
CAN-601451	Carex brunnescens subsp. brunnescens. *Saarela, Gillespie, Sokoloff & Bull 2407.*	http://dx.doi.org/10.6084/m9.figshare.1408640
CAN-598595	*Eriophorum brachyantherum*. *Gillespie, Saarela, Doubt, Bull & Sokoloff 9485.*	http://dx.doi.org/10.6084/m9.figshare.1408555
CAN-598605	*Eriophorum brachyantherum*. *Gillespie, Saarela, Doubt, Bull & Sokoloff 9673.*	http://dx.doi.org/10.6084/m9.figshare.1408553
CAN-598607	*Eriophorum brachyantherum*. *Gillespie, Saarela, Doubt, Bull & Sokoloff 9899.*	http://dx.doi.org/10.6084/m9.figshare.1408554
CAN-598924	*Eriophorum brachyantherum*. *Gillespie, Saarela, Doubt, Bull & Sokoloff 9982.*	http://dx.doi.org/10.6084/m9.figshare.1408557
CAN-598596	*Eriophorum brachyantherum*. *Gillespie, Saarela, Doubt, Bull & Sokoloff 10091.*	http://dx.doi.org/10.6084/m9.figshare.1408556
CAN-598910	*Eriophorum brachyantherum*. *Gillespie, Saarela, Doubt, Bull & Sokoloff 10102.*	http://dx.doi.org/10.6084/m9.figshare.1408558
CAN-598598	*Eriophorum brachyantherum*. *Gillespie, Saarela, Doubt, Bull & Sokoloff 10305.*	http://dx.doi.org/10.6084/m9.figshare.1408561
CAN-592326	*Luzula wahlenbergii*. *Gillespie, Saarela, Consaul & Bull 8170.*	http://dx.doi.org/10.6084/m9.figshare.1408559
CAN-601427	*Triglochin palustris*. *Saarela, Gillespie, Sokoloff & Bull 2535.*	http://dx.doi.org/10.6084/m9.figshare.1408560
CAN-601426	*Triglochin palustris*. *Saarela, Gillespie, Sokoloff & Bull 2652.*	http://dx.doi.org/10.6084/m9.figshare.1408562
CAN-592381	*Corallorhiza trifida*. *Gillespie, Saarela, Consaul & Bull 8093.*	http://dx.doi.org/10.6084/m9.figshare.1408563
CAN-601648	*Corallorhiza trifida*. *Saarela, Gillespie, Sokoloff & Bull 1970.*	http://dx.doi.org/10.6084/m9.figshare.1408564
CAN-601649	*Corallorhiza trifida*. *Saarela, Gillespie, Sokoloff & Bull 2036.*	http://dx.doi.org/10.6084/m9.figshare.1408565
CAN-601650	*Corallorhiza trifida*. *Saarela, Gillespie, Sokoloff & Bull 2415.*	http://dx.doi.org/10.6084/m9.figshare.1408567
CAN-601651	Platanthera obtusata subsp. obtusata. *Saarela, Gillespie, Sokoloff & Bull 2197.*	http://dx.doi.org/10.6084/m9.figshare.1408568
CAN-601276	Platanthera obtusata subsp. obtusata. *Saarela, Gillespie, Sokoloff & Bull 2209.*	http://dx.doi.org/10.6084/m9.figshare.1408566
CAN-601652	Platanthera obtusata subsp. obtusata. *Saarela, Gillespie, Sokoloff & Bull 2488.*	http://dx.doi.org/10.6084/m9.figshare.1408570
CAN-601348	Calamagrostis stricta subsp. groenlandica. *Saarela, Gillespie, Sokoloff & Bull 2191.*	http://dx.doi.org/10.6084/m9.figshare.1408569
CAN-601345	Calamagrostis stricta subsp. groenlandica. *Saarela, Gillespie, Sokoloff & Bull 2255.*	http://dx.doi.org/10.6084/m9.figshare.1408571
CAN-601347	Calamagrostis stricta subsp. groenlandica. *Saarela, Gillespie, Sokoloff & Bull 2398.*	http://dx.doi.org/10.6084/m9.figshare.1408572
CAN-601346	Calamagrostis stricta subsp. groenlandica. *Saarela, Gillespie, Sokoloff & Bull 2442.*	http://dx.doi.org/10.6084/m9.figshare.1408573
CAN-601344	Calamagrostis stricta subsp. groenlandica. *Saarela, Gillespie, Sokoloff & Bull 2576.*	http://dx.doi.org/10.6084/m9.figshare.1408575
CAN-601368	Hordeum jubatum subsp. jubatum. *Saarela, Gillespie, Sokoloff & Bull 2737.*	http://dx.doi.org/10.6084/m9.figshare.1408577
CAN-601369	Hordeum jubatum subsp. jubatum. *Saarela, Gillespie, Sokoloff & Bull 2755.*	http://dx.doi.org/10.6084/m9.figshare.1408576
CAN-432022	Leymus innovatus subsp. velutinus. *Kuc 405.*	http://dx.doi.org/10.6084/m9.figshare.1408579
CAN-432023	Leymus innovatus subsp. velutinus. *Kuc 405.*	http://dx.doi.org/10.6084/m9.figshare.1408580
CAN-601371	Leymus mollis subsp. mollis. *Saarela, Gillespie, Sokoloff & Bull 2529.*	http://dx.doi.org/10.6084/m9.figshare.1408581
CAN-600906	*Puccinellia banksiensis*. *Gillespie, Saarela, Consaul & Bull 7549.*	http://dx.doi.org/10.6084/m9.figshare.1408582
CAN-592678	*Puccinellia banksiensis*. *Gillespie, Saarela, Consaul & Bull 8055.*	http://dx.doi.org/10.6084/m9.figshare.1408585
CAN-592239	*Puccinellia banksiensis*. *Gillespie, Saarela, Consaul & Bull 8055-2.*	http://dx.doi.org/10.6084/m9.figshare.1408583
CAN-592679	*Puccinellia banksiensis*. *Gillespie, Saarela, Consaul & Bull 8077.*	http://dx.doi.org/10.6084/m9.figshare.1408586
CAN-592688	*Puccinellia banksiensis*. *Gillespie, Saarela, Consaul & Bull 8146-2.*	http://dx.doi.org/10.6084/m9.figshare.1408584
CAN-592705	*Puccinellia banksiensis*. *Gillespie, Saarela, Consaul & Bull 8240.*	http://dx.doi.org/10.6084/m9.figshare.1408589
CAN-592689	*Puccinellia banksiensis*. *Gillespie, Saarela, Consaul & Bull 8261.*	http://dx.doi.org/10.6084/m9.figshare.1408588
CAN-592707	*Puccinellia banksiensis*. *Gillespie, Saarela, Consaul & Bull 8339.*	http://dx.doi.org/10.6084/m9.figshare.1408587
CAN-592375	*Stuckenia vaginata*. *Gillespie, Saarela, Consaul & Bull 8048.*	http://dx.doi.org/10.6084/m9.figshare.1408591
CAN-592376	*Suaeda calceoliformis*. *Gillespie, Saarela, Consaul & Bull 7570.*	http://dx.doi.org/10.6084/m9.figshare.1408590
CAN-593265	*Suaeda calceoliformis*. *Gillespie, Saarela, Consaul & Bull 8068.*	http://dx.doi.org/10.6084/m9.figshare.1408592
CAN-593267	*Suaeda calceoliformis*. *Gillespie, Saarela, Consaul & Bull 8137.*	http://dx.doi.org/10.6084/m9.figshare.1408593
CAN-598332	*Suaeda calceoliformis*. *Gillespie, Saarela, Doubt, Bull & Sokoloff 9662.*	http://dx.doi.org/10.6084/m9.figshare.1408594
CAN-598331	*Suaeda calceoliformis*. *Gillespie, Saarela, Doubt, Bull & Sokoloff 10243.*	http://dx.doi.org/10.6084/m9.figshare.1408595
CAN-599149	*Arenaria humifusa*. *Gillespie, Saarela, Doubt, Bull & Sokoloff 9882.*	http://dx.doi.org/10.6084/m9.figshare.1408596
CAN-599166	*Arenaria humifusa*. *Gillespie, Saarela, Doubt, Bull & Sokoloff 9893.*	http://dx.doi.org/10.6084/m9.figshare.1408598
CAN-599167	*Arenaria humifusa*. *Gillespie, Saarela, Doubt, Bull & Sokoloff 9971.*	http://dx.doi.org/10.6084/m9.figshare.1408597
CAN-592340	*Arenaria longipedunculata*. *Gillespie, Saarela, Consaul & Bull 7721.*	http://dx.doi.org/10.6084/m9.figshare.1408599
CAN-593142	*Arenaria longipedunculata*. *Gillespie, Saarela, Consaul & Bull 8136.*	http://dx.doi.org/10.6084/m9.figshare.1408602
CAN-601731	*Arenaria longipedunculata*. *Saarela, Gillespie, Sokoloff & Bull 2477.*	http://dx.doi.org/10.6084/m9.figshare.1408601
CAN-601732	*Arenaria longipedunculata*. *Saarela, Gillespie, Sokoloff & Bull 2776.*	http://dx.doi.org/10.6084/m9.figshare.1408600
CAN-592334	*Sabulina stricta*. *Gillespie, Saarela, Consaul & Bull 7966.*	http://dx.doi.org/10.6084/m9.figshare.1408604
CAN-592360	*Andromeda polifolia*. *Gillespie, Saarela, Consaul & Bull 8002.*	http://dx.doi.org/10.6084/m9.figshare.1408603
CAN-601935	*Andromeda polifolia*. *Saarela, Gillespie, Sokoloff & Bull 2186.*	http://dx.doi.org/10.6084/m9.figshare.1408605
CAN-592359	Orthilia secunda subsp. obtusata. *Gillespie, Saarela, Consaul & Bull 8036.*	http://dx.doi.org/10.6084/m9.figshare.1408606
CAN-601915	Orthilia secunda subsp. obtusata. *Saarela, Gillespie, Sokoloff & Bull 2489.*	http://dx.doi.org/10.6084/m9.figshare.1408608
CAN-598345	Oxytropis deflexa subsp. foliolosa. *Gillespie, Saarela, Doubt, Bull & Sokoloff 10129.*	http://dx.doi.org/10.6084/m9.figshare.1408607
CAN-601898	Oxytropis deflexa subsp. foliolosa. *Saarela, Gillespie, Sokoloff & Bull 2504.*	http://dx.doi.org/10.6084/m9.figshare.1408609
CAN-601901	Oxytropis deflexa subsp. foliolosa. *Saarela, Gillespie, Sokoloff & Bull 2530.*	http://dx.doi.org/10.6084/m9.figshare.1408611
CAN-601900	Oxytropis deflexa subsp. foliolosa. *Saarela, Gillespie, Sokoloff & Bull 2658.*	http://dx.doi.org/10.6084/m9.figshare.1408610
CAN-601899	Oxytropis deflexa subsp. foliolosa. *Saarela, Gillespie, Sokoloff & Bull 2714.*	http://dx.doi.org/10.6084/m9.figshare.1408612
CAN-592385	*Pinguicula vulgaris*. *Gillespie, Saarela, Consaul & Bull 7718.*	http://dx.doi.org/10.6084/m9.figshare.1408613
CAN-592384	*Pinguicula vulgaris*. *Gillespie, Saarela, Consaul & Bull 8132.*	http://dx.doi.org/10.6084/m9.figshare.1408614
CAN-601974	*Pinguicula vulgaris*. *Saarela, Gillespie, Sokoloff & Bull 2264.*	http://dx.doi.org/10.6084/m9.figshare.1408617
CAN-601972	*Pinguicula vulgaris*. *Saarela, Gillespie, Sokoloff & Bull 2381.*	http://dx.doi.org/10.6084/m9.figshare.1408615
CAN-601970	*Pinguicula vulgaris*. *Saarela, Gillespie, Sokoloff & Bull 2478.*	http://dx.doi.org/10.6084/m9.figshare.1408619
CAN-601973	*Pinguicula vulgaris*. *Saarela, Gillespie, Sokoloff & Bull 2531.*	http://dx.doi.org/10.6084/m9.figshare.1408618
CAN-601975	*Pinguicula vulgaris*. *Saarela, Gillespie, Sokoloff & Bull 2565.*	http://dx.doi.org/10.6084/m9.figshare.1408620
CAN-601971	*Pinguicula vulgaris*. *Saarela, Gillespie, Sokoloff & Bull 2787.*	http://dx.doi.org/10.6084/m9.figshare.1408621
CAN-599229	*Pinguicula vulgaris*. *Gillespie, Saarela, Doubt, Bull & Sokoloff 9878.*	http://dx.doi.org/10.6084/m9.figshare.1408623
CAN-599258	*Pinguicula vulgaris*. *Gillespie, Saarela, Doubt, Bull & Sokoloff 9880.*	http://dx.doi.org/10.6084/m9.figshare.1408622
CAN-599230	*Pinguicula vulgaris*. *Gillespie, Saarela, Doubt, Bull & Sokoloff 9967.*	http://dx.doi.org/10.6084/m9.figshare.1408624
CAN-601976	*Utricularia ochroleuca*. *Saarela, Gillespie, Sokoloff & Bull 2464.*	http://dx.doi.org/10.6084/m9.figshare.1408625
CAN-601987	*Primula egaliksensis*. *Saarela, Gillespie, Sokoloff & Bull 2606.*	http://dx.doi.org/10.6084/m9.figshare.1408626
CAN-601986	*Primula egaliksensis*. *Saarela, Gillespie, Sokoloff & Bull 2640.*	http://dx.doi.org/10.6084/m9.figshare.1408629
CAN-602059	*Coptidium × spitsbergense*. *Saarela, Gillespie, Sokoloff & Bull 2194.*	http://dx.doi.org/10.6084/m9.figshare.1408627
CAN-602060	*Coptidium × spitsbergense*. *Saarela, Gillespie, Sokoloff & Bull 2419.*	http://dx.doi.org/10.6084/m9.figshare.1408628
CAN-592250	*Salix arctophila*. *Gillespie, Saarela, Consaul & Bull 7511.*	http://dx.doi.org/10.6084/m9.figshare.1408630
CAN-601675	*Salix fuscescens*. *Saarela, Gillespie, Sokoloff & Bull 2361.*	http://dx.doi.org/10.6084/m9.figshare.1408631
CAN-601674	*Salix fuscescens*. *Saarela, Gillespie, Sokoloff & Bull 2362.*	http://dx.doi.org/10.6084/m9.figshare.1408632
CAN-605793	*Saxifraga eschscholtzii*. *Edlund 41.*	http://dx.doi.org/10.6084/m9.figshare.1408633
CAN-592397	Saxifraga rivularis subsp. arctolitoralis. *Gillespie, Saarela, Consaul & Bull 8174.*	http://dx.doi.org/10.6084/m9.figshare.1408634
